# Evolution of protein kinase substrate recognition at the active site

**DOI:** 10.1371/journal.pbio.3000341

**Published:** 2019-06-24

**Authors:** David Bradley, Pedro Beltrao

**Affiliations:** European Molecular Biology Laboratory, European Bioinformatics Institute (EMBL-EBI), Wellcome Genome Campus, Cambridge, United Kingdom; Yale University, UNITED STATES

## Abstract

Protein kinases catalyse the phosphorylation of target proteins, controlling most cellular processes. The specificity of serine/threonine kinases is partly determined by interactions with a few residues near the phospho-acceptor residue, forming the so-called kinase-substrate motif. Kinases have been extensively duplicated throughout evolution, but little is known about when in time new target motifs have arisen. Here, we show that sequence variation occurring early in the evolution of kinases is dominated by changes in specificity-determining residues. We then analysed kinase specificity models, based on known target sites, observing that specificity has remained mostly unchanged for recent kinase duplications. Finally, analysis of phosphorylation data from a taxonomically broad set of 48 eukaryotic species indicates that most phosphorylation motifs are broadly distributed in eukaryotes but are not present in prokaryotes. Overall, our results suggest that the set of eukaryotes kinase motifs present today was acquired around the time of the eukaryotic last common ancestor and that early expansions of the protein kinase fold rapidly explored the space of possible target motifs.

## Abstract 2

The combined use of phylogenetics, phosphoproteomics, and kinase specificity models reveal that most serine/threonine protein kinase specificities at the active site arose early during the evolution of eukaryotic protein kinases, around the time of the last eukaryotic common ancestor, LECA.

## Introduction

Protein kinases are essential for signal transduction and have been found in every eukaryotic species so far examined. They are required for almost all cellular processes [1], and mutations in protein kinases are often associated with diseases such as cancer and diabetes [2–4]. Kinases are often described in terms of their ‘specificity’, which refers to the set of substrates that the kinase is able to phosphorylate in vivo. Multiple factors define the specificity of the kinase [5]. The kinase and substrate must be coexpressed and colocalised, for example, and their interaction may be mediated by adaptor or scaffold proteins [6,7]. Docking sites on the substrate may also be employed to recruit the kinase and the substrate directly [8,9]. Fundamentally, selectivity is often defined by the structural interface between the kinase active site and the residues flanking the target serine, threonine, or tyrosine—the so-called peptide specificity of the kinase.

The kinase peptide specificity is usually described in terms of a short linear motif [10,11]. The substrate motif of PKA (protein kinase A), for example, is R-R-x-S/T, meaning that an arginine is preferred 2 and 3 positions N terminal to the target serine/threonine in the PKA active site. Conceptually, different substrate motifs can be thought of as different channels of communication within the cell, allowing for kinases that are simultaneously active to regulate a specific set of substrates. Mitotic kinases with overlapping localisations, for example, tend to have mutually exclusive substrate motifs, presumably to prevent the aberrant phosphorylation of nontargets during cell-cycle progression [12].

It is possible that the range of selectivities at the active site is restricted by the structure of the kinase domain itself. In turn, the capacity of the kinase fold to create novel specificity preferences through mutations may determine the maximum effective number of kinases possible for a genome, because it has been suggested for transcription factors [13]. In this analogy to a communication channel, the full set of possible substrate motifs can be thought of as the full bandwidth or ‘communication potential’ of the kinase fold. Understanding how this communication potential was explored over evolutionary time can reveal insights into the evolution of cell pathways and cell signalling. However, although the proliferation of the kinase domain itself has been well documented, much less is known about the evolution of new kinase specificities at the active site [14]. One study found that the frequency of tyrosine kinases (TKs) in the proteome correlates negatively with the frequency of tyrosine residues in the proteome, implying some extent of coevolution between kinases and substrates [15]. Another study found that the evolution of a new specificity in the CMGC (cyclin-dependent kinases (CDKs), mitogen-activated protein kinases (MAP kinases), glycogen synthase kinases (GSK) and CDK-like kinases (CLKs)) group proceeded through an intermediate of broad specificity (P + 1/R + 1) before later specialisation into distinct target preferences (P + 1 and R + 1) [16].

Currently, the scarcity of kinase-substrate interaction data outside of a few model organisms (human, mouse, and budding yeast) is limiting for further research. However, other sources of data can yield insights more indirectly. An evolutionary analysis of the kinase domain can be informative provided that the specificity-determining positions (SDPs) are known [17]. This applies to phosphoproteome data also, provided that motifs can be extracted and linked to the known specificities of kinase families or subfamilies. Here, we collect kinase sequence data, kinase specificity data, and phosphorylation data from several species to perform an evolutionary analysis of kinase specificity. Collectively, the results suggest that most specificities arose early in the evolution of protein kinases, followed by a long period of relative stasis.

## Results

### Global kinase phylogeny for 9 different species

The eukaryotic protein kinase superfamily by convention is divided hierarchically at the level of 'groups', 'families', and 'subfamilies' [18,19]. The most up-to-date classification is based primarily upon sequence similarity between kinase domains but also takes into account the kinase sequence outside of the kinase domain, the known function(s) of the kinase, and previous manual classifications of known orthologs [19,20].

The 8 canonical kinase groups (AGC [PKA, PKG, PKC], CAMK [Calcium- and Calmodulin-regulated kinases], CK1 [Casein Kinase 1], CMGC [cyclin-dependent kinases (CDKs), mitogen-activated protein kinases (MAP kinases), glycogen synthase kinases (GSK) and CDK-like kinases (CLKs)], RGC [Receptor Guanylate Cyclases], STE [sterile mutant], TKL [Tyrosine Kinase-Like], and TK) evolved the earliest and, with the exception of TKs and the RGC group, are thought to have arisen in an early eukaryotic ancestor [21]. Kinase families, and then subfamilies, generally emerged later during evolution and reflect more distinct features of the kinase's function (specificity, regulation, localisation, etc.) [18]. In order to study the evolution of kinase specificity, we first performed a systematic phylogenetic analysis to predict kinase functionally divergent residues for every kinase family and subfamily where possible. To this end, a global kinase domain phylogeny was constructed for the 9 manually curated opisthokont kinomes (*Homo sapiens*, *Mus musculus*, *Strongylocentrotus purpuratus*, *Drosophila melanogaster*, *Caenorhabditis elegans*, *Amphimedon queenslandica*, *Monosiga brevicollis*, *Saccharomyces cerevisiae*, and *Coprinopsis cinerea*) present in the kinase database KinBase (http://kinase.com/web/current/kinbase/).

**Fig 1:**
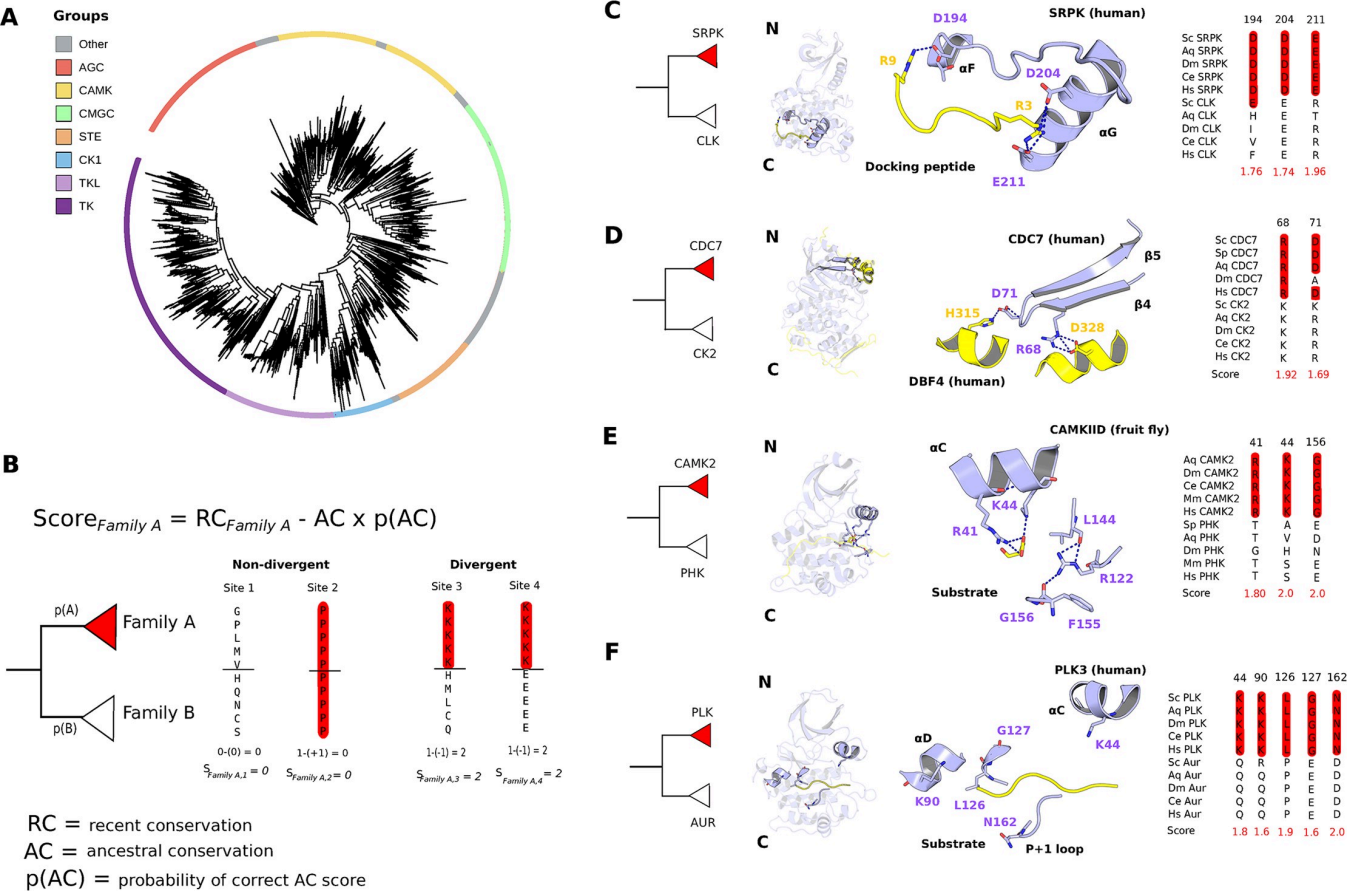
Family and subfamily divergent residues. (A) Kinase domain phylogeny for 2,094 different kinase domains, spanning 9 species. Each sequence has been coloured according to its group membership. (B) Explanation of the score used to identify divergent residues. RC: conservation of the residue within the family of interest. AC: extent to which the ancestral residues are conserved between sister clades in the phylogeny. p(AC): a measure of confidence in the ancestral sequence prediction. A full explanation is given in the Materials and methods section. (C–F) Examples of residues predicted to be functionally divergent in the SRPK (PDB: 1WBP), CDC7 (PDB: 4F9A), CAMK2 (PDB: 5H9B), and PLK (PDB: 4B6L with peptide binding modelled) families. In these 4 examples, kinase residues have been numbered according to their position in the protein kinase domain (Pfam: PF00069). The peptides and/or proteins that physically interact with the kinase have been coloured in yellow. AC, ancestral conservation; AGC, (PKA, PKG, PKC); AUR, Aurora Kinase; CAMK, Calcium- and Calmodulin-regulated kinases; CAMK2, Calcium/calmodulin-dependent protein kinase type 2; CDC7, Cell Division Cycle 7-related protein kinase; CK1, Casein Kinase 1; CK 2, Casein Kinase 2; CLK, CDK-Like Kinase; CMGC, (cyclin-dependent kinases (CDKs), mitogen-activated protein kinases (MAP kinases), glycogen synthase kinases (GSK) and CDK-like kinases (CLKs)); PDB, Protein Data Bank; PHK, Phosphorylase *K*inase; Pfam, Protein families; PLK,; Polo-like kinase RC, recent conservation; SRPK, SR-rich protein kinase; STE, sterile mutant; TK, tyrosine kinase; TKL, Tyrosine Kinase-Like.

The resulting phylogeny contains 2,094 kinase sequences spanning 8 different groups (Fig 1A). Although based just on the kinase domain sequence, the grouping of kinase sequences accords well with the group/family/subfamily classifications provided by KinBase. For example, out of 102 families tested, 70 show exact correspondence to the KinBase classifications in that all family members group together to the exclusion of kinase sequences from any other family (i.e., the family is monophyletic). This was found to be the case also for 69 out of 105 subfamily kinases tested. For the remaining families and subfamilies tested (32 and 36, respectively), we consider for further analysis (described below) only the largest clade containing the family/subfamily sequences of interest. These contain relatively few ‘spurious’ sequences on average from other families and subfamilies (median clade purity 91.5% and 66.7%, respectively) and tend to cover a large proportion of all kinases annotated by KinBase to a particular family or subfamily (median family coverage: 95.1%, median subfamily coverage: 100%).

### Residues implicated in the differentiation of duplicated kinases

For 99 kinase families and 83 kinase subfamilies, we identified residues that are conserved within a clade but differ from the sister clade in the phylogeny. These residues are implicated as functionally divergent residues and are expected to underlie functional differences between kinase sister clades. This was achieved by calculating divergence scores (s) for each alignment position and each family and subfamily using an adaptation of the phylogenetic BADASP (Burst After Duplication with Ancestral Sequence Predictions) method [22]. Among other methods [23–26], we selected BADASP given that it enables ancestral family sequences to be compared directly (Fig 1B; Materials and methods), allowing determinant positions arising at a specific point in time to be captured using user-defined protein family definitions [22].

Functionally divergent residues were first predicted across all kinase families. A detailed analysis of the results suggests multiple ways in which novel kinase functions have evolved at the family level via changes to functionally relevant residues. In the SRPK (SR-rich protein kinase) family (CMGC), for example, 2 substitutions to negatively charged amino acids (D and/or E) map to parts of the kinase structure (αF-αG region) that have been shown previously to bind to a positively charged docking peptide (Fig 1C; [27]). In the CDC7 (Cell Division Cycle 7-related protein kinase) family (CMGC) also, 2 of the identified functionally divergent residues bind to the CDC7 activator protein named Dbf4 (Dumbbell former 4 protein) (Fig 1D; [28]) and are therefore important for kinase regulation. In other examples, the functionally divergent residues identified can help to account for the specificity of the kinase. Two substitutions in the CAMK2 family (CAMK), for example, bind to a preferred D/E residue at the substrate +2 position (PDB [Protein Data Bank]: 5H9B). A glycine substitution in the activation loop has also been shown to be important for kinase function [29] and may explain why CAMK2 kinases do not require activation loop phosphorylation for activity ([30], Fig 1E). Finally, many substitutions for the acidophilic PLK (Polo-like kinase) family map to SDRs (specificity-determining residues)and are convergent with those identified for the unrelated GRK(G-protein-coupled Receptor Kinase) family (AGC), which contains some acidophilic kinases ([17,31], Fig 1F). These examples illustrate how the predicted functionally divergent sites between families or subfamilies of kinases can map to functionally relevant residues. We next studied whether this would be a general feature of these residues across many families and subfamilies.

### Functionally important residues are often divergent across kinase families and subfamilies

**Fig 2:**
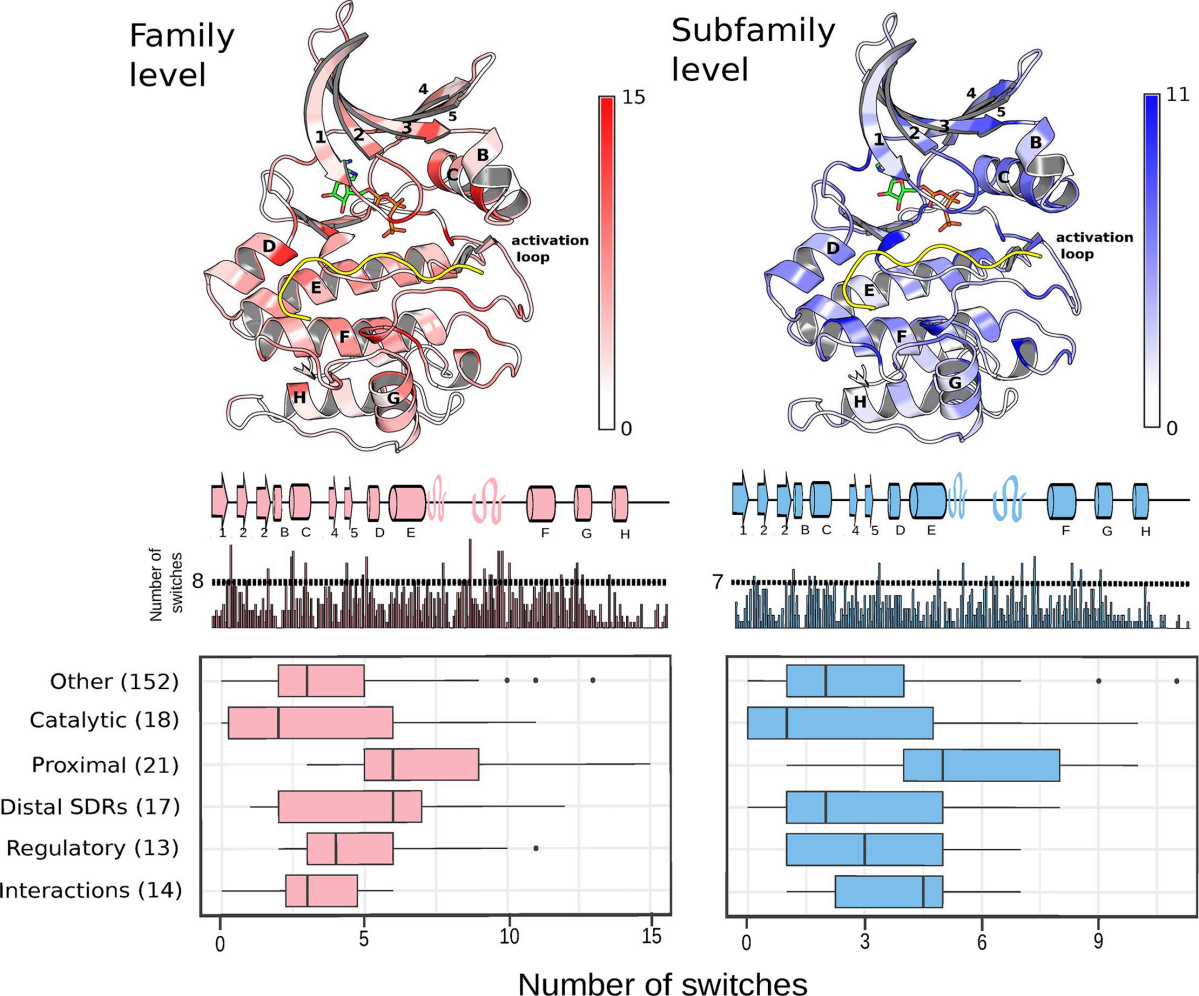
Aggregated analysis of sequence divergence across kinase families (left) and subfamilies (right). For each kinase domain position, the total number of ‘switches’ (score > 95th percentile of scores) was counted across all families or subfamilies considered (see Materials and methods). (Top) Results mapped to kinase structures (mouse protein kinase A: PDB 1ATP). The kinases are represented in complex with an ATP molecule (green, orange, blue, red) and a substrate-mimicking inhibitor (PKIA, yellow). Darker shades of red or blue represent a higher number of switches (see colour bar, right). (Middle) Total number of switches mapped to the kinase primary sequence, with secondary structure elements represented above the bar plot. A domain position is considered to be ‘frequently switching’ if the number of switches lies above a 90th percentile threshold for the kinase domain. The threshold is 8 for families and 7 for subfamilies. (Bottom) The values for each domain position have been grouped according to the functional category (‘catalytic’, ‘regulatory’, ‘proximal’, etc.) and the distribution plotted separately at the family and subfamily level. The numbers in brackets represent the number of residues in each category. PDB, Protein Data Bank; PKIA, cAMP-dependent protein kinase inhibitor alpha; SDR, specificity-determining residue.

Across all kinase families, we aggregated the total number of predicted functionally divergent residues or ‘switches’ at each position in the kinase domain. This allows us to predict positions that often determine the functional differences between kinase families. These were mapped across the kinase catalytic domain sequence and fold (Fig 2, left). The distribution of residues that are often implicated in kinase family functional differences is not uniform and strongly enriched within or close to the kinase activation segment, the αC helix, the β5-αD region, and the αF-αG regions (Fig 2, left). For further analysis, we divided kinase residues into functional categories: ‘catalytic’ (catalytic residues and the catalytic spine), ‘proximal’ (within 4 Angstroms of the peptide substrate but excluding ‘catalytic’ positions), ‘distal SDRs’ (distal SDRs implicated in [17]), ‘regulatory’ (regulatory spine residues and those within and surrounding the activation loop), ‘interaction’ residues (those most frequently in contact with other protein domains), and ‘other’ (residues not belonging to any of the previous categories). All residues belonging to these categories are defined in S1 Table, using mappings to a PKA reference (PDB: 1ATP) and the Pfam (Protein families) protein kinase domain (Pfam: PF00069). We defined as frequently switching residues those above the 90th percentile of residues with most changes. The majority (14/21) of frequently switching residues can be assigned to a functional category (catalysis, specificity, regulation, etc.), which is more than would be expected by chance (*p* = 0.0048; Fisher's exact test, one-sided). This suggests that this approach can successfully identify residues that are of relevance for the functional divergence of kinases. Of these residues, 8 have been implicated in determining differences in kinase specificity: domain position 84 [32–34], 86 [35], 144 [16,36,37], 157 [37,38], 158 [35,37], 162 [35], 164 [39,40], and 205 [35]. The number of substitutions for ‘proximal’ residues is generally higher than that for residues without an assigned function (Mann-Whitney, one-tailed, *p* = 3.2 × 10^−6^). These residues lie at the kinase-substrate interface and are likely to perturb kinase specificity when mutated. We find similar results when using SDR annotations from a global analysis of kinase specificity [41] (Mann-Whitney, one-tailed, *p* = 9.0 × 10^−3^) and when using a literature-curated set of SDRs provided by the same study [41] (Mann-Whitney, one-tailed, *p* = 3.6 × 10^−5^).

A similar analysis was performed for kinase subfamily comparisons (Fig 2, right). Similar to kinase family evolution, a large fraction (73%, 11 out of 15) of residues frequently implicated in the functional differences between kinase subfamilies were also annotated to a functional category (*p* = 0.0043; Fisher's exact test, one-sided). All 15 frequently switching residues have been mapped to the protein kinase fold in S1 Fig, alongside the 21 frequently switching residues at the family level. We also observed a higher than expected number of switches for ‘proximal’ residues at the subfamily level when compared with residues not annotated with a function (Mann-Whitney, one-tailed, *p* = 2.2 × 10^−5^). This was found to be the case also when using the SDR annotations in [41] (Mann-Whitney, one-tailed, *p* = 2.2 × 10^−4^) and a set of literature-curated SDRs from the same study (Mann-Whitney, one-tailed, *p* = 1.7 × 10^−3^).

All switch events within the ‘proximal’ category at the family and subfamily levels have been mapped to the kinase phylogeny in S2 Fig. Taken together, these results suggest that substrate-determining residues often undergo substitutions as new kinase families and subfamilies emerge. We note, however, that the probability that any given residue in the ‘proximal’ category will diverge between sister families is relatively low; the most frequently substituted residue was found to have switched in only 17.6% of families analysed, for example (S3 Fig). The relationship between kinase sequence divergence and kinase specificity divergence is discussed further below.

### Evolution of experimentally determined kinase target preferences

The above results suggest that residues important for kinase specificity differ often across kinase families and subfamilies. We then studied the extent to which these changes in kinase residues impact upon their target specificity. To study this, we derived specificity models for 101 S/T kinases from human and mouse, using experimentally determined target sites listed in the literature-curated databases HPRD (Human Protein Reference Database), Phospho.ELM, and PhosphoSitePlus [42–44]. This approach tends to produce PWMs (position weight matrices) that are similar to those generated using a peptide-screening approach [35].

**Fig 3:**
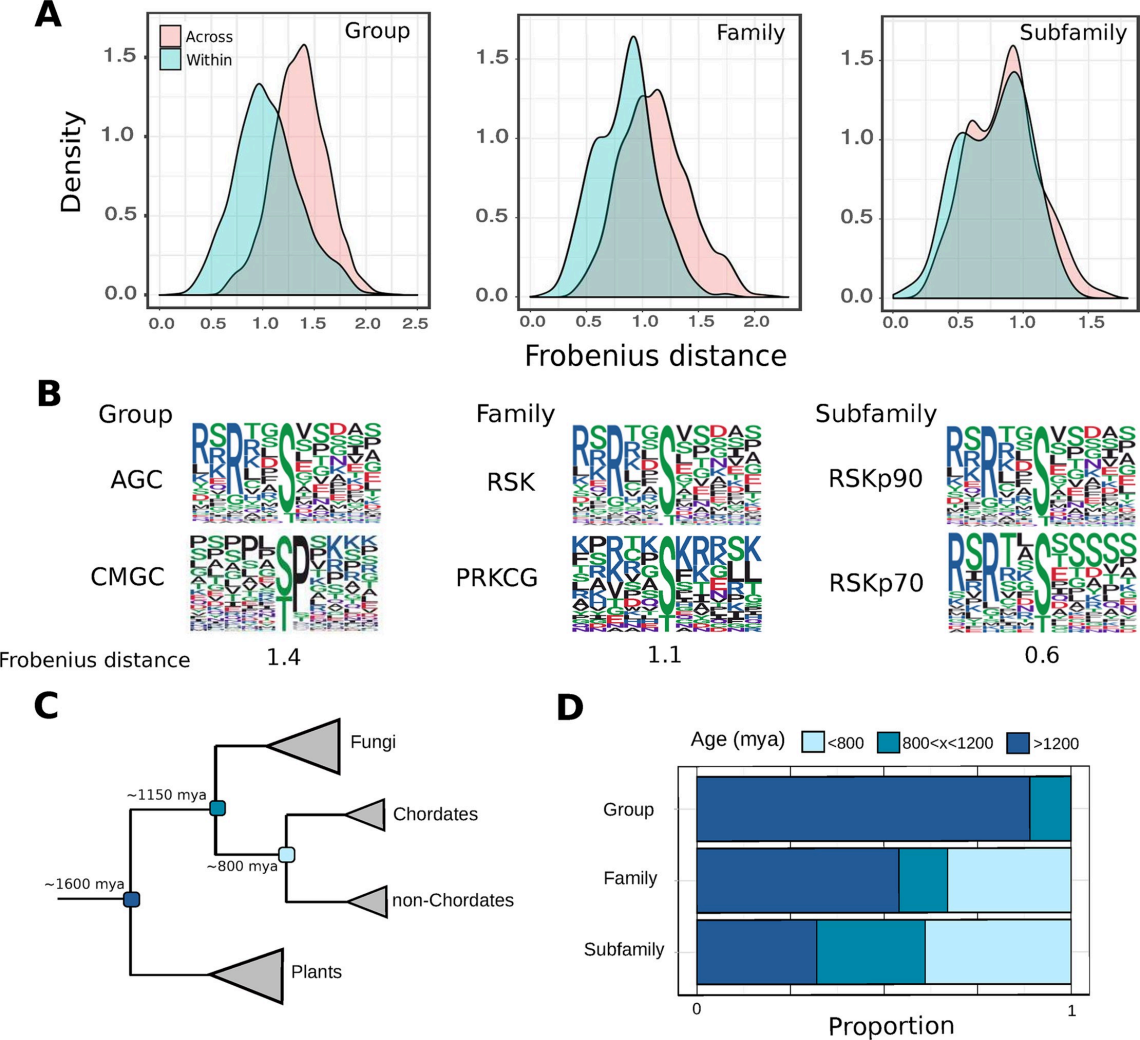
Evolution of kinase specificity at the group, family, and subfamily level. (A) Differences in S/T kinase specificity models at the group, family, and subfamily levels. The Frobenius distance was calculated for all possible pairwise comparisons within and between groups, families, and subfamilies. (B) Representative kinase pairs belonging to different groups (left), families (centre), and subfamilies (right). Frobenius distances for each of the 3 pairs are given beneath the logos. (C) A simplified tree of life with 3 important divergence times (plant-opisthokont, fungi-metazoa, chordate-nonchordate) marked. (D) Phylogenetic estimation of kinase ages at the group, family, and subfamily level for S/T kinases. AGC, (PKA, PKG, PKC); CMGC, cyclin-dependent kinases (CDKs), mitogen-activated protein kinases (MAP kinases), glycogen synthase kinases (GSK) and CDK-like kinases (CLKs); mya, million years ago; PRKCG, Protein Kinase C gamma; RSK, Ribosomal S6 Kinases; RSKp70, Ribosomal S6 Kinases p70; RSKp90, Ribosomal S6 Kinases p90; S/T, serine/threonine.

We then tested the extent to which kinase specificities differ within and between groups, families, and subfamilies for kinases of known specificity. In line with expectation, the differences in specificity are larger across groups than across families and also larger across families than across subfamilies (Fig 3A). For subfamilies, the differences are not statistically different from the distances measured within subfamilies (*p* = 0.19, Kolmogorov-Smirnov test, two-sided). These results suggest that kinase specificity often diverges at the level of the group, less so at the family level, and rarely when new subfamilies emerge. We show in Fig 3B some examples of typical differences in kinase specificity for the 3 classifications. Although the differences in specificity across families is statistically different from expectation (*p* ≪ 0.01, Kolmogorov-Smirnov test, two-sided), the ‘typical’ differences observed are smaller than at the group level. This is illustrated by the RSK (Ribosomal S6 Kinases) and PKC (Protein Kinase C) families (Fig 3B, centre), which both have a preference for arginine at the −3 position, but PKC additionally has a preference for R at position +2 whereas RSK has a modest preference for the same residue at position −5.

We next considered the relationship between the divergence in kinase sequence and divergence in kinase specificity. Even for kinase sequences of unknown function (‘other’ category, *n* = 152), the sequence identity between kinase pairs was found to correlate with the distances between their PWMs (r = −0.39, *p* ≪ 0.01). The correlation between the two was even greater for residues of the ‘proximal’ category (*n* = 21, r = −0.48, *p* ≪ 0.01) and for a ‘high-confidence’ set of residues (*n* = 10, r = −0.48, *p* ≪ 0.01; S5 Fig) close to the substrate that covary strongly with specificity [17]. We then considered the extent of sequence change required before divergence in specificity could be observed, which we define here using a PWM Frobenius distance of 1.06 (see Materials and methods). Generally, for the ‘other’, ‘proximal’, and ‘high-confidence SDR’ categories, new specificities emerge as sequence identities fall below approximately 55%, approximately 70%, and approximately 80%, respectively (S5 Fig), corresponding to 68, 6, and 2 required switches, respectively. These data suggest that specificity changes at the family and subfamily level should be relatively rare, because only 9 kinase families and 4 kinase subfamilies were found with at least 6 ‘proximal’ switch residues. However, we note that the impact of residue mutation will likely depend upon the nature of the substitution (‘conservative’ or ‘nonconservative’), the substrate position affected (e.g., +1 and −3 tend to be much more important for specificity than −1), and the existence of compensatory mutations in the kinase domain that might buffer the effect of SDR mutations, because other studies have demonstrated experimentally that a single SDR mutation can be sufficient to radically alter specificity [36,38,40].

To put the previous results into the context of evolutionary time scales, we sought to date the emergence of many kinase groups, families, and subfamilies. To this end, the presence or absence of every S/T kinase group, family, and subfamily was predicted for several species across the tree of life (Fig 3C). The phylogenetic origin of kinase groups, families, and/or subfamilies was then predicted using ancestral state reconstructions, which allowed their emergence to be dated based on the known divergence times between species [45]. Overall, we estimated that most kinase groups arose in a universal eukaryotic ancestor, in line with a previous study [21]. For kinase families, around 55% are estimated to have arisen in a universal ancestor, and up to 65% have arisen before the split between chordates and nonchordates (approximately 800 million years ago [mya]). Around 60% of subfamilies were similarly estimated to have arisen before the split between chordates and nonchordates (Fig 3D). Together with the analysis of kinase specificity differences, this result suggests that relatively few kinase specificities are likely to have arisen in the past 800 million years of kinase evolution.

### Kinase motif enrichment across 48 eukaryotic species

The analysis of kinase specificity differences described above can only be performed for kinases with many experimentally determined targets. For most kinases, this information is not available [17,46]. As an alternative way to study the evolution of kinase specificity, we analysed MS (mass spectrometry)-derived phosphorylation sites from a broad range of species. The phosphoproteome of any given species represents an ensemble of kinase activities. Many of these kinases will have preferred target site sequence motifs that are required for optimal substrate phosphorylation. The signature of several different kinases may therefore be encoded in each phosphoproteome.

**Fig 4:**
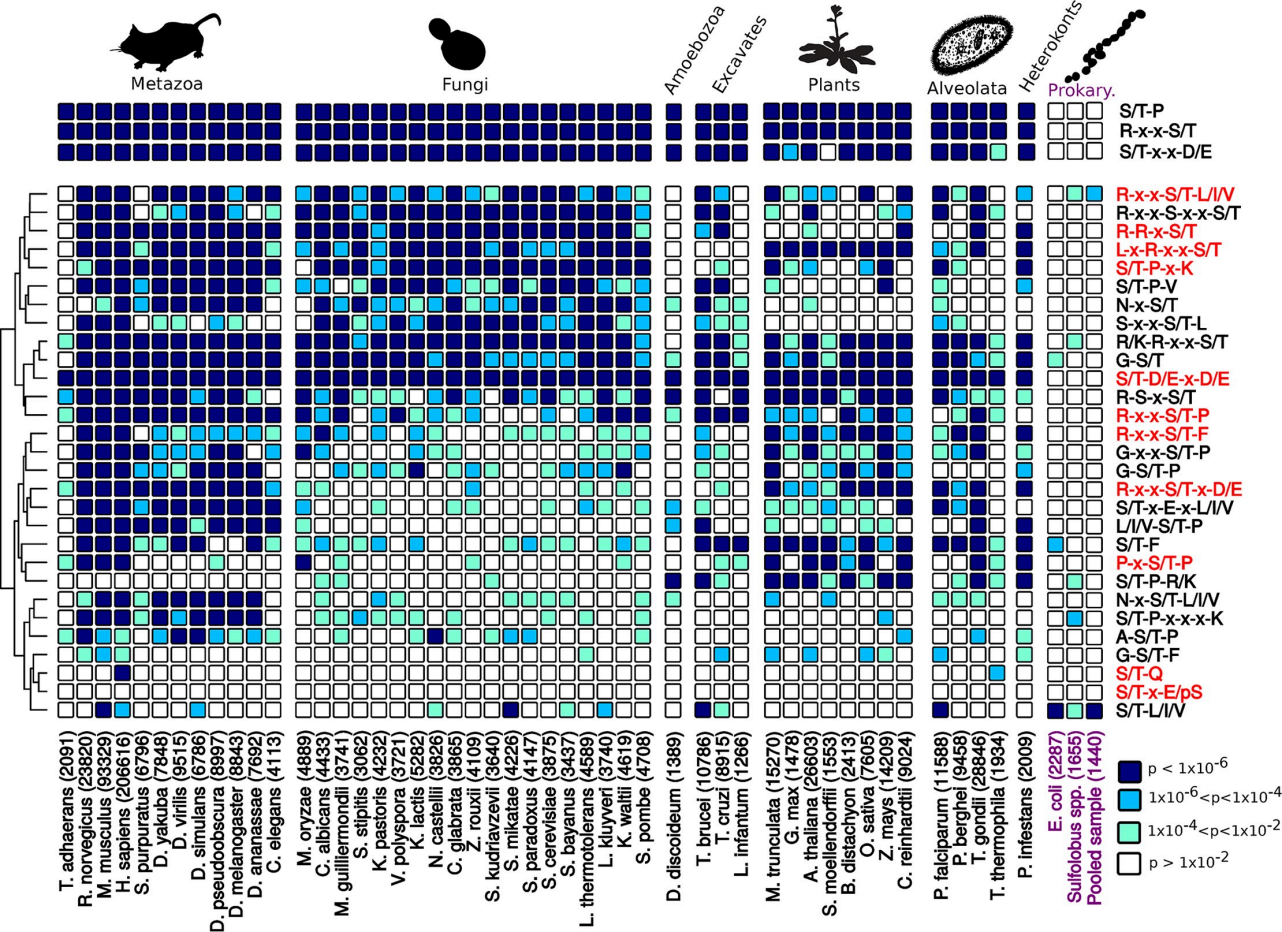
Enrichment of S/T phosphorylation motifs across several species. Binomial *p*-values were calculated for each motif and each species considered. The heat map cells are coloured according to the extent of enrichment for that particular motif and species (see legend, bottom right). The numbers in the column labels correspond to the sample size of unique S/T phosphorylation sites (15-mer). Prokaryotic phosphosite samples are coloured in purple. (Top) Enrichment of 3 common phosphorylation signatures (S/T-P, R-x-x-S/T, and S/T-x-x-D/E). (Bottom) Enrichment of 29 motifs discovered using the motif-x tool. Motifs in which the effector kinase has already been described in the literature are coloured in red. S/T, serine/threonine.

For this study, we were interested in determining the extent to which different kinase-substrate motifs have been exploited during the evolution of the eukaryotes. To this end, phosphoproteome data were collected from 48 eukaryotic species, including species from the alveolates (4), amoebozoa (1), excavates (3), fungi (19), heterokonts (1), metazoa (12), and plants (8). We first measured the enrichment of 3 well-established substrate signatures (R-x-x-S/T, S/T-P, and S/T-x-x-D/E) and found them to be strongly enriched in nearly all of the 48 species (Fig 4, top). This suggests that these 3 common preferences are likely to have been present very early on during the evolution of the eukaryotes. To extend this to other kinase preferences, target site S/T sequence motifs were extracted from each species phosphoproteome using the motif-x tool [47]. Motifs without consistent enrichment across related species were filtered from any further analysis (see Materials and methods). In total, 29 motifs were (Fig 4) identified, which account for approximately 54% of all phosphosites analysed (S6A Fig).

Among the 29 motifs identified, 11 have been characterised previously in the literature and have been assigned to at least 1 kinase family or subfamily. This includes well-known motifs such as the CDK (Cyclin-Dependent Kinase) family motif (S/T-P-X-K) and the CK2 family motif (S/T-D/E-x-D/E). Eight of the motifs feature either a proline at position +1 or an arginine at position −3. Other motifs were identified in addition to those that are well characterised (Fig 4, motifs in black). Here, multiple constraints were imposed to ensure that the selected motifs were likely to represent bona fide kinase target motifs. For example, motifs with simple S/T additions to a classical motif were filtered from the analysis, because they could result from phosphosite misassignment within phosphopeptides from the mass spectrometry analysis or potential clustering of phosphorylation sites in the substrate primary sequences [48,49]. Overall, 18 uncharacterised motifs were selected using the protocol described in the Materials and methods section. Some of these motifs feature ‘new’ substrate specificity determinants such as asparagine (N) and glycine (G).

In Fig 4, enrichment *p*-values for each motif were calculated for each species relative to a background set of shuffled phosphorylation site sequences, with the S/T retained at the centre. This analysis suggests that the majority of motifs (Fig 4) are pervasive across the eukaryotic tree of life. This finding is even more evident when phosphorylation data are pooled from each of the major taxonomic clades (animals, fungi, plants, etc.) and the enrichment *p*-values are recalculated (S6B Fig and S6C Fig). Most of the motifs analysed are distributed across clades that diverged early during the evolution of the eukaryotes. For example, 18 out of 29 motifs (62%) are highly enriched (*p* < 1 × 10^−6^) in animals, fungi, and plants, indicating that they are likely to be of ancient origin.

The distribution of motif enrichments between related species is nonrandom, as supported by tests for the phylogenetic signal of phosphorylation motifs (S2 Table). We tested whether kinase motif enrichments correlate with the frequency of their expected effector kinases in the kinome, including, for example, the frequency of CDKs with the frequency of S/T-P-x-K motifs. However, our analysis suggests that kinase family or subfamily frequencies are not generally correlated with motif enrichment values when the phylogenetic interdependence of data points is taken into account [50] (S7 Fig). In spite of this, there is local evidence of kinase-substrate coevolution for some clades and motifs. In the plants, for example, the lack of enrichment of the basophilic R-R-x-S/T motifs can likely be explained by the depletion of its cognate effector kinase (PKA/PKG (Protein Kinase G)) (S8 Fig), as has been suggested previously [51,52]. For the CDK family also, the pattern of S/T-P-x-K evolution and CDK evolution is similar across many species (S9 Fig). However, many other patterns cannot be similarly accounted for, which suggests that there are multiple factors that can affect the fold enrichment values calculated.

### Kinase motif enrichment in prokaryotes

Some kinases encoded in the genome of prokaryotes are homologous to eukaryotic protein kinases and are currently referred to as ePK-like kinases (ELKs) [53,54]. Current genomic data now suggest that these eukaryotic-like kinases are as prevalent as histidine kinases in the prokaryotes [55]. Until recently however, the S/T phosphoproteomes of archaean and bacterial species had remained poorly characterised [56]. We repeated the motif analysis on the species *Escherichia coli* (bacteria) and *Sulfolobus* spp. (archaea), which are currently the only 2 organisms with more than 1,000 determined S/T phosphosites [56–58]. This analysis suggests that a large majority of the eukaryotic phosphorylation motifs discussed previously are not significantly enriched in these 2 species (Fig 4). For *E*. *coli*, there is a moderate enrichment for some single-site motifs, which could have evolved convergently with those motifs found in eukaryotes. For *Sulfolobus*, we observe only weak enrichment for 5 eukaryotic motifs (S/T-L/I/V, S/T-P-x-x-x-K, S/T-P-R/K, R/K-R-x-x-S/T, and R-x-x-S/T-L/I/V). We note also that the S/T-P and R-x-x-S/T motifs that are highly prevalent in the eukaryotes show no evidence of enrichment across the prokaryotic species tested (Fig 4). Although it could be argued that the low phosphosite sample sizes (2,287 and 1,655 for *E*. *coli* and *Sulfolobus*, respectively) precludes the reliable detection of weaker motifs, we note that both of these signatures (S/T-P and R-x-x-S/T) were found to be strongly enriched in eukaryotic species with small sample sizes (*Dictyostelium discoideum*: 1,389, *Leishmania infantum*: 1,266). A similar lack of enrichment was found for a pooled sample of prokaryotic phosphorylation sites (*n* = 1,440) from several species [57] (Fig 4).

Prokaryotic motifs were then identified de novo using the motif-x tool for *E*. *coli* and the *Sulfolobus* genus. None of the motifs identified overlap with the motifs recovered previously for eukaryotic species (S3 Table and S4 Table). For *Sulfolobus*, in particular, we also found that 5 out of the 7 motifs identified contain a positively charged residue (R/K), implying the existence of basophilic *Sulfolobus* kinases.

## Discussion

Here, we have explored the evolution of protein kinase specificity at the active site, using a combination of kinase sequence data, phosphorylation data, and kinase specificity models. Using the sequence of protein kinases across several species, we have shown that the evolution of new kinase families is dominated by sequence changes that are likely to impact upon kinase function, including kinase peptide specificity. This is in line with our observation that kinases belonging to different groups and families typically show significant differences in their specificity. In contrast, kinases belonging to sister subfamilies do not show significant differences in their specificity. A phylogenetic analysis revealed that most kinase groups and families (89% and 54%, respectively) are of ancient origin among the eukaryotes, whereas subfamilies generally emerged later during evolution (only 32% are of ancient origin). Finally, phosphorylation motifs determined for 48 eukaryotic species were found to be broadly distributed across divergent species and likely emerged soon before or after the last eukaryotic common ancestor. Taking these different observations together, we suggest that the majority of the kinase active site specificities present today in eukaryotic species have emerged early on during the evolution of eukaryotes.

The analysis here of divergent residues across kinase families follows a similar analysis employing a BLAST-based approach [59]. This study is focused on the kinase catalytic domain, and we did not take into account the evolution of kinase domain composition or sequence changes outside the catalytic domain, which may have a significant impact on catalytic function [60]. Kinase docking interfaces also are not amenable to the aggregated analysis attempted here because their location in the kinase domain tends to differ significantly between families [8]. In general, the approach used here assumes that a given kinase domain position will adopt a given function (catalytic, regulatory, proximal, etc.) across all kinase families. However, examples are known already of modes of regulation or specificity that are particular to a given family or subfamily [61,62]. The important residues may be functionally misannotated in such cases, which would underestimate the extent of divergence in regulatory or substrate-specific functions. Such kinase-specific examples of residue function may account for many of the switching events currently placed in the ‘other’ category.

From the analysis of kinase specificity models, it is apparent that new specificities are often generated following the emergence of a new kinase group or family, but not following the emergence of a new kinase subfamily. This is not a surprising result given that kinase groups and families tend to be older than kinase subfamilies (Fig 3D). We note, however, that the sample size of PWMs for this analysis was relatively low (*n* = 101) and that many of the kinase targets gathered from in vitro studies tend to lack strong in vivo support [44]. The approach used for PWM construction relies on literature-curated targets sites and therefore tends to be biased towards medically relevant and/or well-studied kinases [63,64]. However, more systematic approaches have recently been developed [40,65,66], which may in the future enable the characterisation of many kinases in parallel, in a similar vein to a 2010 specificity-based study of 61 *S*. *cerevisiae* kinases [35].

The observation that subfamilies from the same family tend to have the same specificity is, however, in conflict with the finding that SDR substitutions are also present throughout the evolution of subfamilies (Fig 2, subfamilies). There are 2 known cases in the literature (PLK and GRK) in which moderate differences in specificity are observed between sister subfamilies [31,67–69]. We suggest that differences in peptide specificity can exist between subfamilies, but on average and based on the current small sample size of specificity models, these tend to be modest at the subfamily level.

Finally, the analysis of phosphorylation motifs across 48 different eukaryotic species suggests that most arose either in an early eukaryotic ancestor or shortly before the emergence of the last eukaryotic common ancestor. The phosphorylation data set spans the tree of life but is unsurprisingly biased towards animal, fungal, and plant species. Ongoing projects for increased representation of the protist superkingdoms could help to address this problem in the future [70]. From this analysis, we also conclude that most eukaryotic phosphomotifs post-date the divergence of eukaryotes and prokaryotes. The acquisition of phosphoproteome data from several more prokaryotic species will be required, however, to strengthen this conclusion. In general, the increase in statistical power enabled by larger data sets will enable the reliable identification of weakly enriched motifs (‘false negatives’), many of which are likely missing from this analysis. However, this would not eliminate the possibility that the phosphomotifs observed may reflect the specificities of phospho-binding domains such as the WW domain, Polo-box domain, and 14-3-3 domains [71,72]. A more definitive analysis would require the acquisition of direct kinase-substrate relationship data across several species.

Collectively, the results suggest that the evolution of new kinase specificities was characterised by a ‘burst’ around the time of the last eukaryotic common ancestor, followed by a period of relative stasis. Most gene duplicates will be quickly silenced [73], and diversification of function is often considered a primary means for the ‘survival’ of newly duplicated genes in the genome. The capacity of the kinase fold to generate diverse target preferences at the active site interface through mutations may have been an important factor underlying the success of this fold. Our analysis suggests that, over the past 800 million years, there have been relatively few novel motifs emerging in eukaryotic kinases. It is interesting to speculate why this is the case. It is possible that no new distinct mode of interaction can be accommodated at the active site or that such novel motifs are not easily reached via mutations of existing kinases. As mentioned above, kinase specificity is determined via multiple mechanisms, including docking interactions, expression differences, localisation differences, activation modes, etc. Duplicated kinases can, therefore, be made nonredundant by diversifying the way by which they regulate their substrates to avoid misregulation in multiple different ways [12]. Additional research will be needed to study how the different kinase specificity mechanisms have evolved in kinases.

Protein kinases are just one of many peptide-binding domain types that can recognize diverse sets of peptide motifs. Other such domains include, for example, the PDZ (Post-synaptic density protein 95 (PSD-95), *Drosophila* disc large tumor suppressor (Dlg1), Zona occludens 1 (ZO-1)), SH2 (Src Homology 2), SH3 (Src Homology 3), and WW, among many other families. It remains to be seen whether the findings described here relating to the evolution of different target motifs will apply to other such important peptide-binding domains.

## Materials and methods

### Kinase phylogenetic analysis

Kinase domain sequences were collected for all 9 opisthokont species in KinBase with an annotated kinome (*H*. *sapiens*, *M*. *musculus*, *S*. *purpuratus*, *D*. *melanogaster*, *C*. *elegans*, *A*. *queenslandica*, *M*. *brevicollis*, *S*. *cerevisiae*, and *C*. *cinerea*) [19]. Kinases of the ‘atypical’ group were excluded from the analysis. The kinase domain sequences were then aligned using MAFFT [74], were filtered to remove pseudokinases (kinases without expected residues at domain positions 30, 48, 123, 128, and 141), and then were realigned using MAFFT L-INS-i [74]. Manual corrections were then made to the multiple sequence alignment (MSA), and the trimAl tool was employed to remove positions with 20% or more of 'gap' characters among the sequences [75]. Finally, a further filter was applied to remove truncated sequences with fewer than 190 kinase domain positions.

The resulting MSA (2,094 sequences) was used to generate a maximum-likelihood kinase domain phylogeny with the RaxML tool [76]. Amino acid substitutions were modelled using the LG matrix, and a gamma model was employed to account for the heterogeneity of rates between sites. A neighbour-joining phylogeny generated with the R ape package was used as the starting tree [77].

Ancestral sequence reconstructions were performed with the CodeML program (part of the PAML package) using an LG substitution matrix [78]. No molecular clock was assumed (clock = 0), and a gamma model was employed again to account for rate heterogeneity between sites. The alpha parameter of the gamma distribution was estimated (fix_alpha = 0) with a staring value of 0.5 (alpha = 0.5), and 4 categories of the gamma distribution were specified (ncatG = 4). The physicochemical properties of the amino acids were not taken into account when performing the ancestral sequence reconstructions (aaDist = 0).

For the analysis of kinase evolution, each family and subfamily was assessed iteratively, and a divergence score (s) was assigned to each position of the MSA. The divergence scores are calculated by comparing the family/subfamily of interest (clade A) with the closest sister clade (clade B) in the phylogeny. The score calculated is adapted from the BADX score of a previous publication by Edwards and colleagues [22], specifically:

$$S={RC}_{A}-{AC}_{x}.\;p(AC).$$

Recent conservation (RC) represents the sequence conservation for the clade of interest (clade A) and is calculated here on the basis of substitution matrix similarity in the R package bio3d [79]. AC_X_ represents the conservation of ancestral nodes for the clade of interest (clade A) and the ancestral node for the nearest sister clade (clade B); this is given as a 1 if the predicted residues are identical to each other and a −1 otherwise. Finally, the score is weighted by the value p(AC), which represents the probability that the AC value was correctly assigned. For matching residues (AC = 1), this is the posterior probability of the predicted residue for clade B; for differing residues (AC = −1), this is the summed posterior probability of all residues in clade B besides from the predicted residue for clade A. Therefore, scores for suspected divergence would be down-weighted if there is ambiguity concerning the nature (matching or mismatching) of the clade B ancestor.

Where the sequences of interest were divided into 2 or more clades in the phylogeny, only the largest clade was considered for further analysis. In some cases, also, the clade of interest contained spurious sequences from the wrong family or subfamily. Spurious sequences were tolerated only if they comprised less than 15% of the clade sequences; otherwise, the largest 'pure' subclade (with the sequences of interest only) was selected for further analysis. For the calculation of divergence scores, the nearest sister clade to the clade of interest was selected. However, scores were only calculated if both clades contained 5 or more sequences and belonged to the correct category (e.g., 2 subfamilies that are being compared must belong to the same family). All searching and/or manipulation of the phylogeny was performed using a custom script in R with the aid of the ape package.

### Aggregated analysis across kinase families and subfamilies

For the global analysis represented in Fig 2, the total number of switches for each alignment position was calculated at the family and subfamily levels. For this aggregated analysis, ‘duplicate comparisons’ (i.e., in which the same 2 ancestral nodes were compared) were filtered out to ensure that the same switch event was only counted once. A substitution is considered a switch if it is above the 95th percentile for all subfamily (s_subfamily(95)_ = 1.904) or family (s_family(95)_ = 1.793) scores. For the one-sided Fisher test described in the Results section, a site is considered to be ‘frequently switching’ if the number of switches is above the 90th percentile of switch frequencies for the 246 alignment positions. This was calculated separately at the family (90th percentile = 8) and subfamily (90th percentile = 7) level.

In Fig 2, the aggregated number of switches have been grouped according to the functional categories ‘catalytic’, ‘proximal’, ‘distal’, ‘regulatory’, ‘interactions’, and ‘other’. The ‘catalytic’ residues refer to those that are needed for catalysis or form the catalytic spine [80]. The ‘proximal’ category refers to noncatalytic residues within 4 Angstroms of the substrate peptide of PKA (PDB: 1ATP), excluding substrate positions N terminal to the −6 position. The 'distal SDRs' are the predicted SDRs in Bradley and colleagues [17] more than 4 Angstroms from the substrate (in PDB:1ATP). The 'regulatory' category refers to regulatory spine residues and those within and surrounding the activation loop [81]. The ‘interaction’ category refers to residues often found to be in contact with other protein domains (at least 10) in cocrystal structures, as determined using the 3DID database of protein–protein interactions [82]. Finally, ‘other’ represents any residue not belonging to any of the categories described above. The residues belonging to each category are defined in S1 Table.

When using the SDR annotations from the Creixell and colleagues [41] study, SDRs with a score >0.8 and within 5 Angstroms of the substrate peptide were used for this definition. When using the literature-curated SDRs from the same study, the ‘proximal’ and ‘distal’ categories were merged because only 5 of the literature-curated SDRs were distal from the substrate.

### Analysis of kinases with known specificity

For the analysis of kinase specificity, 101 high-confidence specificity models of human and mouse S/T kinases were collected as described in Bradley and colleagues (2018). These models are derived from the literature-curated kinase targets given in the databases HPRD, Phospho.ELM, and PhosphoSitePlus [42–44]. Each kinase was annotated at the group, family, and subfamily level (as required) using the manual annotations given in the kinase.com website [19]. The analysis of specificity divergence was performed separately at each of the 3 levels. For each level, all pairwise distances within a grouping are computed, and then all possible pairwise distances are calculated between groupings. Importantly, the higher-level categorisation is retained for all pairwise comparisons. For example, at the family level, all between-family distance comparisons would occur for kinases belonging to the same 'group'. For each pairwise comparison, the Frobenius distance between specificity models was calculated using the 'norm(,type = ‘F’) function in R after subtracting one PWM from the other. The Frobenius distance represents the sum of squared differences between matrix values, followed by square rooting [83].

For the comparison of kinase sequence divergence with kinase specificity divergence (S5 Fig), a Frobenius distance threshold of 1.06 was used to separate ‘specificity-conserved’ kinase–kinase pairs from ‘specificity-diverged’ kinase–kinase pairs. This threshold was derived by taking the maximum distance between PWMs generated for the same kinase, which were constructed by subsampling known targets (*n* = 25) from kinases with 50 or more annotated targets.

### Dating the emergence of kinase groups, families, and subfamilies

First, all known kinase groups, families, and subfamilies present in animal and fungal species were retrieved from the kinase database KinBase [19]. The list was then filtered to remove atypical kinases and tyrosine protein kinases. The presence or absence of each kinase group, family, and subfamily across several species of the eukaryotic tree of life was then predicted using the KinAnnote tool [84]. For this purpose, we used all species from a recently published tree of life for which a publicly available genome/proteome sequence was available [85]. The tree of life was then pruned in R using the ape package to retain these species only (55 in total). The origin of each kinase group, family, and subfamily was then predicted using maximum-likelihood–based ancestral state reconstruction with the ace function of the ape package [77]. The reported divergence times between species in the literature was then used to estimate ages for each group, family, and subfamily [45].

Where multiple origins were predicted for a kinase group, family, and subfamily, we traced the kinase emergence to the most recent common ancestral node between the predicted nodes of origin. This approach assumes no horizontal gene transfer between species or convergent evolution of kinase groups, families, and subfamilies.

### Kinase motif enrichment across eukaryotic species

The phosphorylation site data were collected from a range of sources. They are as follows: *Trypanosoma brucei* [86,87], *T*. *cruzi* [88,89], *L*. *infantum* [90], *Trichoplax adhaerens* [91], *H*. *sapiens*/*M*. *musculus*/*Rattus norvegicus* [46], *S*. *purpuratus* [92], *Drosophila* spp. [93], *C*. *elegans* [94], *Magnaporthe oryzae* [95], 18 fungal species [96], *D*. *discoideum* [97], *Medicago truncatula* [98,99], Glycine max [99,100], *Arabidopsis thaliana* [99,101], *Selaginella moellendorffii* [102], *Brachypodium distachyon* [103], *Oryza sativa* [99,104], *Zea mays* [99,105], *Chlamydomonas reinhardtii* [106], *Plasmodium falciparum*/*P*. *berghei*/*Toxoplasma gondii* [107,108], *Tetrahymena thermophila* [109], and *Phytophthora infestans* [51].

For each species, redundant phosphosite 15-mers (centred on S or T) were filtered from the analysis. Phosphorylation motifs (S/T) for each of the 48 species were obtained by running r-motif-x using its default parameters (*p*-value of 1 × 10^−6^ and a minimum of 20 motif occurrences). This tool takes as its input a 'foreground' set of known target sites and a 'background' set of sites known not to be target sites [110]. For the background set, we randomly shuffled the flanking sequences of known phosphorylated target sites (central S/T retained). The amino acid composition of the foreground and background sets were therefore identical. This approach is expected to generate fewer spurious motif predictions than simply sampling S/T sites randomly from the proteome [111]. To generate the background set, each known target site was randomly shuffled 10 times.

For further analysis, we selected only those motifs appearing in at least a third of species within one or more superphyla (i.e., fungi, metazoa, and plants). For the excavates (3 species represented here), the motif had to be present in at least 2 of the examined species. Motifs exclusive to the amoebozoa or heterokonts were not considered because both superphyla are represented here by only a single species. Other constraints were imposed to filter out potentially spurious motifs. Serine or threonine additions to a classical motif were not considered, because they may result from phosphosite misassignment within phosphopeptides or the clustering of phosphorylation sites in the substrate primary sequence [48,49]. We also considered R/K, D/E, and L/I/V/M to be synonymous when identifying new motifs. Finally, D/E additions to the classic casein kinase 2 motif ‘S/T-D/E-x-D/E’ were not considered because weak D/E preferences outside the +1 and +3 positions have already been described for this kinase [38]. Motifs detected here that do not match the list of motifs given in Amanchy and colleagues [112] or Miller and colleagues [113] are declared to be ‘new’ motifs with an unknown upstream regulator.

The enrichment of kinase motifs was calculated relative to the background set of randomised peptides. The significance of motif enrichments in each species was determined by calculating binomial *p*-values. Here, the null probability of the motif is taken to be equal to the total frequency of motif matches (e.g., P-x-S/T-P) to the background set, divided by the total number of background matches for the superset motif (e.g., S/T-P). The calculation of equivalent frequencies for the foreground set enables an analytical *p*-value to be calculated using the binomial distribution. The calculated *p*-value therefore gives an indication, for each motif, of the extent of enrichment of the motif against the background set relative to that of the most frequent superset motif (e.g., the enrichment of P-X-S/T-P relative to S/T-P).

### Number of motif matches as a percentage of the phosphoproteome

Each of the motifs previously identified using motif-x was screened against the known target sites of each species, and the total number of target sites matching at least one motif was counted and then divided by the total number of known target sites in each species (S6A Fig). For this analysis, we do not consider motifs with only one constrained flanking position (e.g., G-S/T), because matches to the foreground set are likely to arise just by chance. These patterns may represent incomplete sequence motifs. Exceptions are made for the classic S/T-P and R-x-x-S/T signatures, which by themselves can be sufficient for kinase targeting [5,114].

### Kinase motif enrichment for prokaryotic phosphorylation sites

The prokaryotic phosphorylation data were collected from multiple sources. Phosphorylation data for *E*. *coli* derives from [56–58]. Phosphorylation data for *Sulfolobus acidocaldarius* and *Sulfolobus solfataricus* comes from the dbPSP database [57]. The pooled species in Fig 4 represents 180 unique phosphorylation sites from 8 prokaryotic species—*Halobacterium salinarum*, *Bacillus subtilis*, *Mycobacterium tuberculosis*, *Streptomyces coelicolor*, *E*. *coli*, *Synechococcus* sp., *S*. *solfataricus*, *S*. *acidocaldarius*—all of which derive from the dbPSP database also [57].

Enrichment values and binomial *p*-values were calculated using the same methods described in the previous section. The motif-x tool was executed using its default parameters, as described above.

### Coevolution between the kinome and phosphoproteome

A starting phylogeny for the 48 eukaryotic species was assembled using the NCBI taxonomy tool [115]. Unresolved branches (polytomies) for particular clades were then resolved manually after referring to previous phylogenetic studies in the literature [116–120]. Kinome annotations for each species were generated automatically using the KinAnnote tool [84], which employs BLAST- and HMM-based searches to identify and classify eukaryotic protein kinases.

The relationship between kinase motifs and their cognate kinases (e.g., S/T-P-x-K and CDKs) was modelled with phylogenetic independent contrasts (PICs) in R using the ape package [50,77]. This method generates phylogenetic contrasts between variables on a tree to account for the nonindependence of data points [50]. In S7 Fig, contrasts were generated for motif enrichment values on the y-axis and for relative kinase frequencies (number of kinases of interest divided by the total number of kinases detected in the proteome) on the x-axis.

Tests for the phylogenetic signal of different motifs were conducted in R using the Phylosignal package [121]. The phylogenetic plots in S8 Fig and S9 Fig were also generated using Phylosignal.

## Supporting information

S1 Fig Frequently switching residues in kinases. Residues coloured in red and blue mark ‘frequently switching’ residues (number of switches above the 90th percentile of switch frequencies across the kinase domain) at the family (A) and subfamily level (B), respectively. The kinases (mouse protein kinase A: PDB 1ATP) are represented in complex with an ATP molecule (green, orange, blue, red) and a substrate-mimicking inhibitor (PKIA, yellow). Kinase residues have been numbered according to their position in the protein kinase domain (Pfam: PF00069). PDB, Protein Data Bank; Pfam, Protein families; PKIA, cAMP-dependent protein kinase inhibitor alpha.
(TIF)

S2 Fig Global kinase domain phylogeny with 2,094 different sequences represented. Red and blue circles represent families and subfamilies (respectively) where divergent residues are found in kinase residues close to the substrate (i.e., the ‘proximal’ category). The size of the circle is proportional to the number of switches found in the ‘proximal’ category.
(TIF)

S3 Fig Relative number of switches for each kinase domain position at the family and subfamily level. Here, the number of switches has been divided (normalised) by the total number of families (*n* = 85) and subfamilies (*n* = 64) considered when aggregating the number of switches. As in Fig 2, the values for each domain position have been grouped according to the functional category (‘catalytic’, ‘regulatory’, ‘proximal’, etc.) of the residues.
(TIF)

S4 Fig Frobenius distances between PWMs generated for the same kinase. On the left (‘same’), PWMs of the same kinase were generated by subsampling all known kinase target sites derived from literature-curated databases. The left-hand box plot represents the distribution of matrix distances between PWMs generated using this same method. The right-hand box plot (‘different’) represents matrix distances between PWMs generated using 2 different methods: phosphosite-based and peptide-screening–based [35]. Only 13 kinases characterised in [35] have sufficient known substrates for PWM construction (therefore, *n* = 13). As expected, PWMs generated using different approaches are more different on average than those generated using the same method. However, in most cases, the inter-PWM distances are comparable to those found for the ‘same’ category. PWM, position weight matrix.
(TIF)

S5 Fig Relation between kinase sequence and specificity differences. (Top) Plot between the kinase sequence identity (x-axis) and Frobenius distance (y-axis) for all possible kinase-kinase pairs among the 101 S/T kinases for which specificity models have been constructed. This has been plotted for residues of the ‘other’ category (left), ‘proximal’ category (centre), and 10 high-confidence SDRs (right) identified in [17]. (Bottom) For the same residue descriptions, plot of kinase sequence identity (x-axis) against the specificity divergence (y-axis). ‘Specificity divergence’ represents the proportion of Frobenius distances above the 1.06 threshold used to separate ‘specificity-conserved’ kinase–kinase pairs from ‘specificity-diverged’ kinase–kinase pairs (see Materials and methods). SDR, specificity determining residue; S/T, serine/threonine.
(TIF)

S6 Fig Motif presence across species. (A) Proportion of phosphorylation sites in each species that match a phosphorylation motif (see Materials and methods). (B) A simplified version of the eukaryotic tree of life presented in the [85] study. The numbers in brackets correspond to the number of different species represented by phosphorylation data in this study. (C) Calculation of binomial *p*-values (as in Fig 4) for each motif in each major clade (metazoa, fungi, plants, etc.) after phosphorylation sites within a clade were pooled across species. The figure legend (bottom right) is the same as in Fig 4.
(TIF)

S7 Fig Phylogenetic independence tests. PICs between 5 different kinase clades (AKT/SGK, CAMK2, CDK, CK2, PKA/PKG) and their corresponding substrate motifs (R-x-x-S/T-F, R-x-x-S/T-x-D/E, S/T-P-x-K, S/T-D/E-x-D/E, and R-R-x-S/T, respectively). This approach accounts for the phylogenetic nonindependence between data points when comparing 2 continuous variables [50]. CAMK2, Calcium/calmodulin-dependent protein kinase type 2; CDK, Cyclin-Dependent Kinase; CK2, Casein Kinase 2; PIC, phylogenetic independence contrast; PKA, Protein Kinase A; PKG, Protein Kinase G; SGK, Serum and Glucocorticoid-regulated Kinase.
(TIF)

S8 Fig Mapping of PKA/PKG family relative kinase frequencies and substrate motif enrichments to a phylogeny of 48 eukaryotic species. Relative kinase frequencies across the 48 species were calculated for the PKA/PKG family, and motif enrichments were calculated for their cognate substrate motif (R-R-x-S/T). The red box highlights species where the absence of PKA and PKG kinases in the proteome corresponds to a lack of R-R-x-S/T motif enrichment. PKA, Protein Kinase A; PKG, Protein Kinase G.
(TIF)

S9 Fig Mapping of CDK family relative kinase frequencies and substrate motif enrichments to a phylogeny of 48 eukaryotic species. Relative kinase frequencies across the 48 species were calculated for the CDK family, and motif enrichments were calculated for its cognate substrate motif (S/T-P-x-K). CDK, Cyclin-Dependent Kinase.
(TIF)

S1 Table A table mapping the kinase MSA used for all sequence analysis (column 1) to the sequence of human protein kinase A (column 2), to the PDB numbering (PDB: 1ATP) of protein kinase A (column 3), and to the kinase domain positions (Pfam: PF00069). MSA, multiple sequence alignment; PDB, Protein Data Bank; Pfam Protein families.
(XLSX)

S2 Table Five tests for the phylogenetic signal (Cmean, I, K, K.star, and Lambda) of 9 different eukaryotic motifs. Numbers in the table represent *p*-values for each one of the tests. Low *p*-values (e.g., *p* < 0. 01) suggest that the motif in question is nonrandomly distributed with respect to the species phylogeny of 48 eukaryotic species (as presented in S3 Fig and S4 Fig). All tests were performed using the Phylosignal package in R [121].
(XLSX)

S3 Table Motifs identified from phosphorylation sites in *E. coli* (*n* = 2,287) using the motif-x tool. In both instances, motif-x was executed using its default parameters (*p* < 1 × 10^−6^ and at least 20 occurrences). The motif-x scores for each of the motifs are displayed in the second column.
(XLSX)

S4 Table Motifs identified from phosphorylation sites in *Sulfolobus* spp. (*n* = 1,655) using the motif-x tool. In both instances, motif-x was executed using its default parameters (*p* < 1 × 10^−6^ and at least 20 occurrences). The motif-x scores for each of the motifs is displayed in the second column.
(XLSX)

## Abbreviations

AC: ancestral conservation
AGC: (PKA, PKG, PKC)
AUR: Aurora Kinase
BADASP: Burst After Duplication with Ancestral Sequence Predictions
CAMK: Calcium- and Calmodulin-regulated kinases
CAMK2: Calcium/calmodulin-dependent protein kinase type 2
CDC7: Cell Division Cycle 7-related protein kinase
CDK: Cyclin-Dependent Kinase
CK1: Casein Kinase 1
CK2: Casein Kinase 2
CLK: CDK-Like Kinase
CMGC: cyclin-dependent kinases (CDKs), mitogen-activated protein kinases (MAP kinases), glycogen synthase kinases (GSK) and CDK-like kinases (CLKs)
Dbf4: Dumbbell former 4 protein
ELK: ePK-like kinase
GRK: G-protein-coupled Receptor Kinases
HPRD: Human Protein Reference Database
MS: Mass spectrometry
MSA: multiple sequence alignment
mya: million years ago
PDB: Protein Data Bank
PDZ: Post-synaptic density protein 95 (PSD-95), Drosophila disc large tumor suppressor (Dlg1), Zona occludens 1 (ZO-1)
Pfam: Protein families
PHK: Phosphorylase Kinase
PIC: phylogenetic independent contrast
PKA: Protein Kinase A
PKC: Protein Kinase C
PKG: Protein Kinase G
PKIA: cAMP-dependent protein kinase inhibitor alpha
PLK: Polo-like kinase
PRKCG: Protein Kinase C gamma
PWM: position weight matrix
RC: recent conservation
RGC: Receptor Guanylate Cyclase
RSK: Ribosomal S6 Kinases
RSKp70: Ribosomal S6 Kinases p70
RSKp90: Ribosomal S6 Kinases p90
SDP: specificity-determining positions
SDR: specificity-determining residue
SGK: Serum and Glucocorticoid-regulated Kinase
SH2: Src Homology 2
SH3: Src Homology 3
SRPK: SR-rich protein kinase
S/T: serine/threonine
STE: sterile mutant
TK: tyrosine kinase
TKL: Tyrosine Kinase-Like
