# Supplementary figures and images for: Evolution of protein kinase substrate recognition at the active site
Source: PLoS Biol. 2019 Jun 24;17(6):e3000341. doi: 10.1371/journal.pbio.3000341 (PMC6611643; doi:10.1371/journal.pbio.3000341)

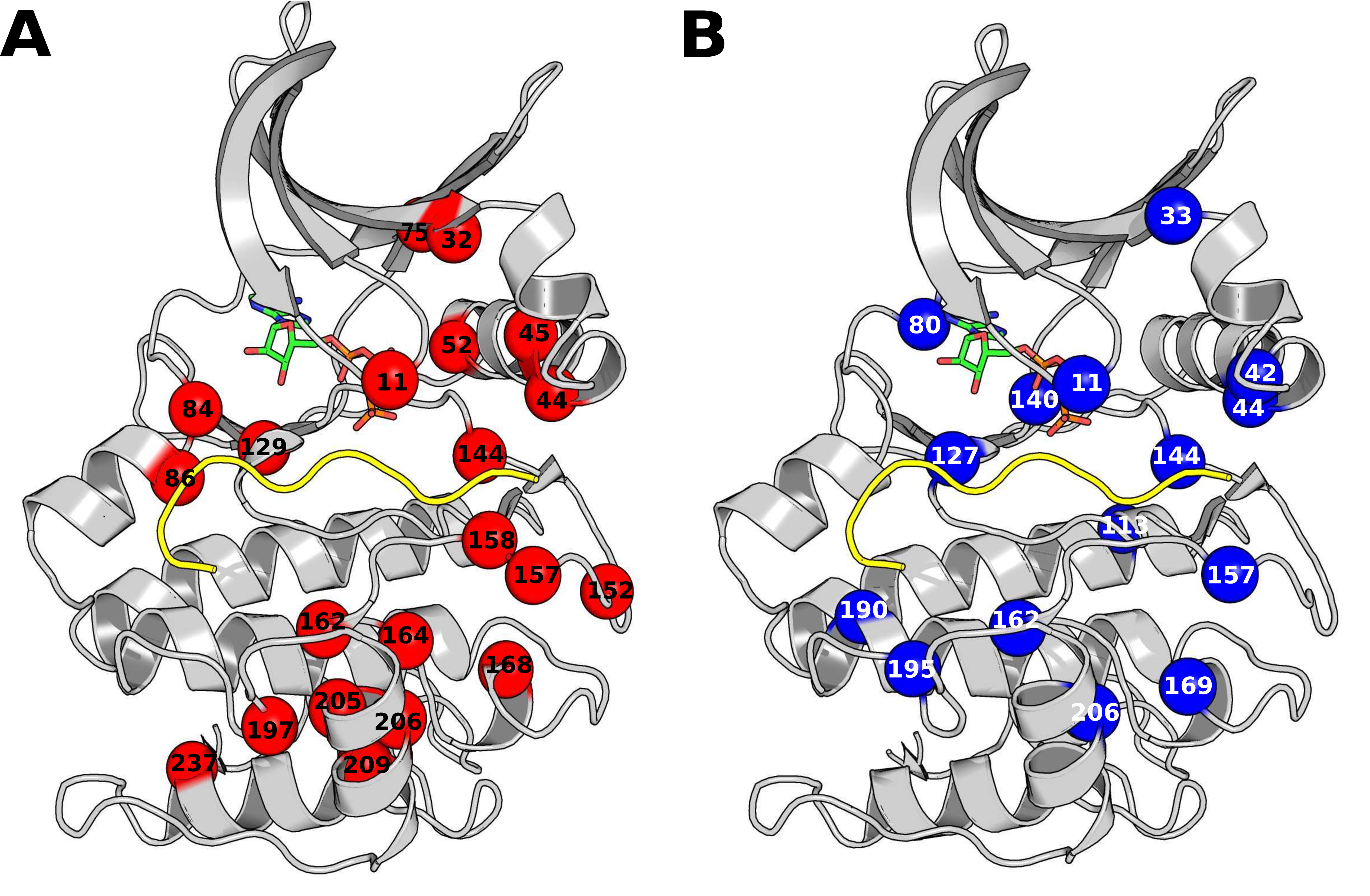

Supplement: S1 Fig — Residues coloured in red and blue mark ‘frequently switching’ residues (number of switches above the 90th percentile of switch frequencies across the kinase domain) at the family (A) and subfamily level (B), respectively. The kinases (mouse protein kinase A: PDB 1ATP) are represented in complex with an ATP molecule (green, orange, blue, red) and a substrate-mimicking inhibitor (PKIA, yellow). Kinase residues have been numbered according to their position in the protein kinase domain (Pfam: PF00069). PDB, Protein Data Bank; Pfam, Protein families; PKIA, cAMP-dependent protein kinase inhibitor alpha. (TIF) [file pbio.3000341.s001.tif]

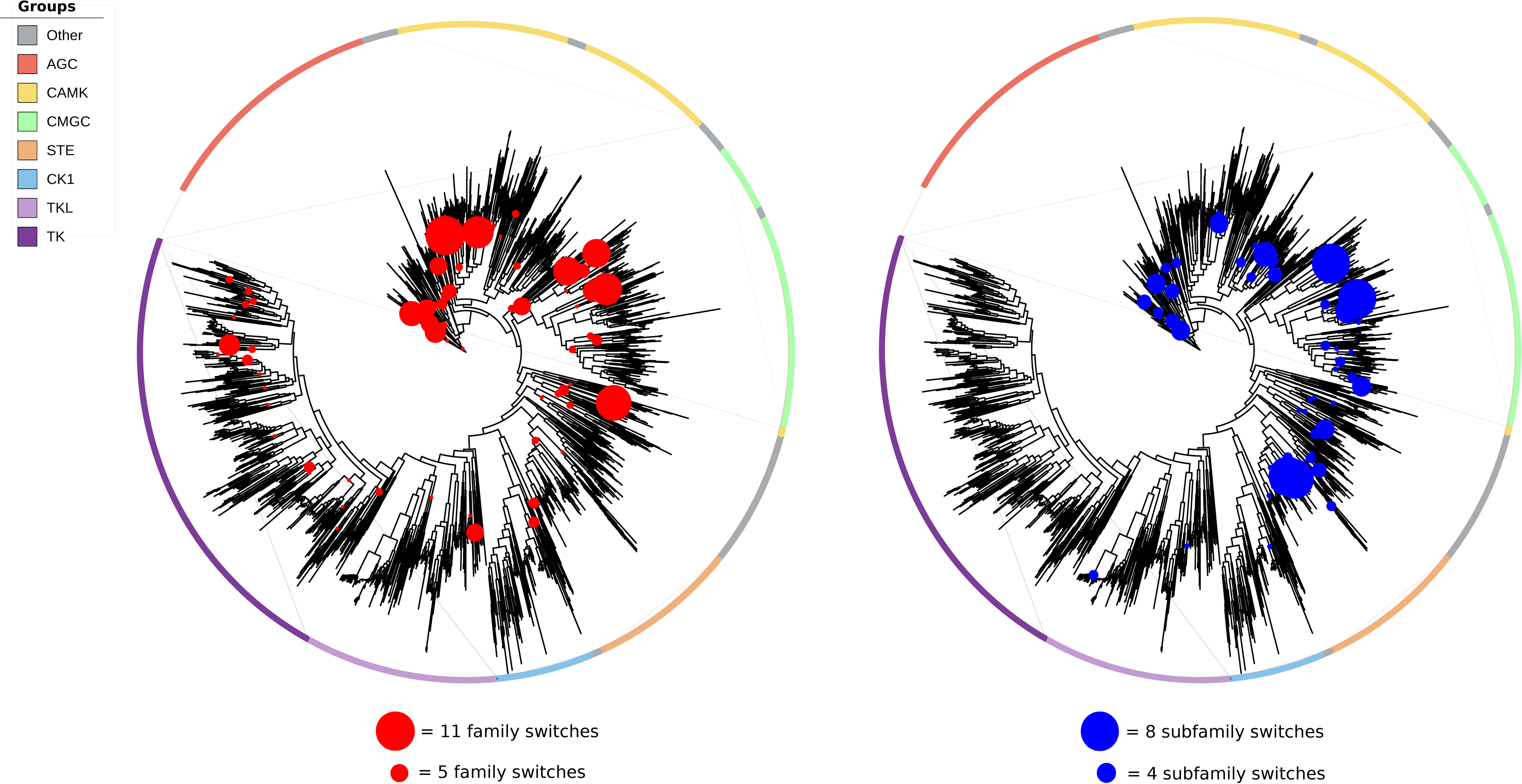

Supplement: S2 Fig — Red and blue circles represent families and subfamilies (respectively) where divergent residues are found in kinase residues close to the substrate (i.e., the ‘proximal’ category). The size of the circle is proportional to the number of switches found in the ‘proximal’ category. (TIF) [file pbio.3000341.s002.tif]

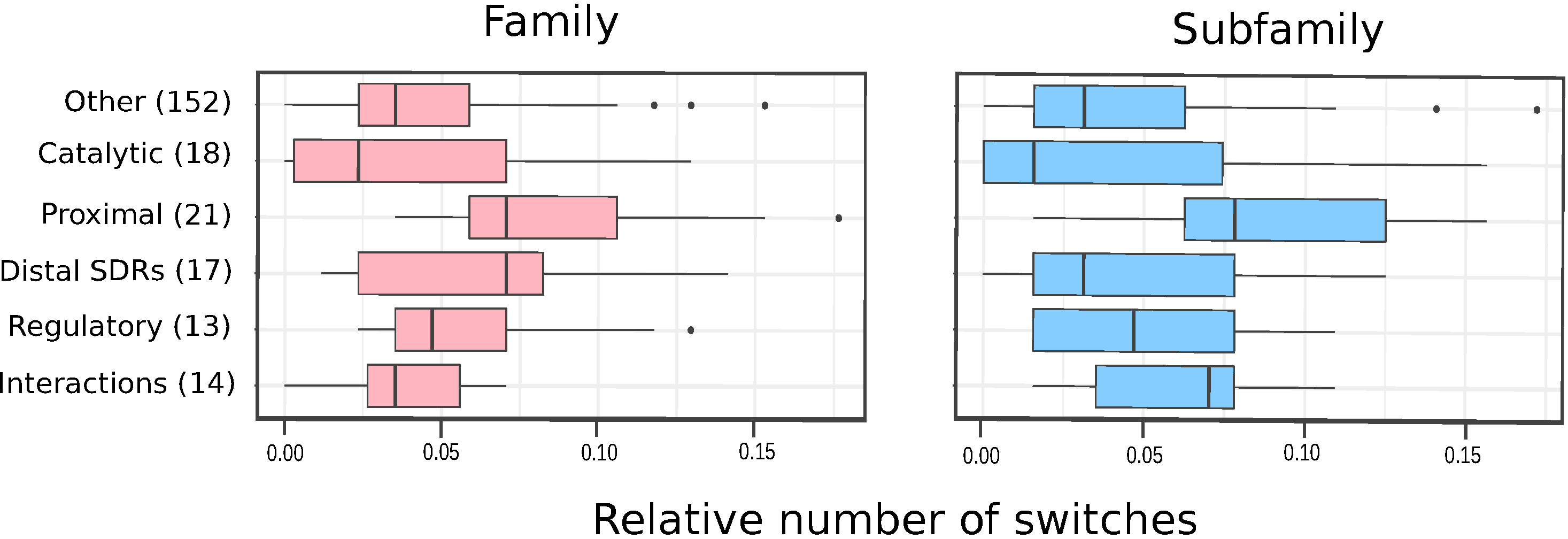

Supplement: S3 Fig — Here, the number of switches has been divided (normalised) by the total number of families (n = 85) and subfamilies (n = 64) considered when aggregating the number of switches. As in Fig 2, the values for each domain position have been grouped according to the functional category (‘catalytic’, ‘regulatory’, ‘proximal’, etc.) of the residues. (TIF) [file pbio.3000341.s003.tif]

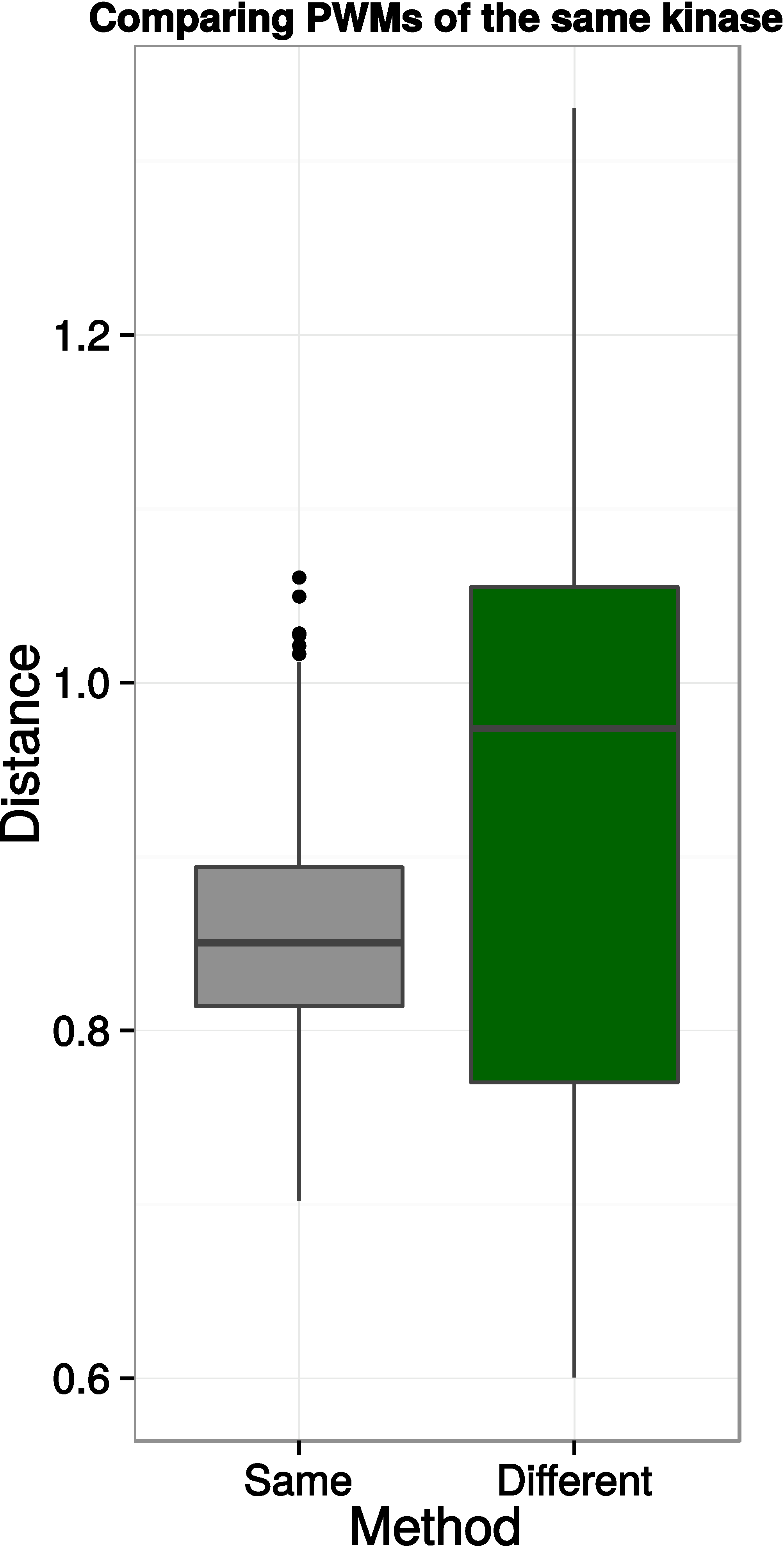

Supplement: S4 Fig — On the left (‘same’), PWMs of the same kinase were generated by subsampling all known kinase target sites derived from literature-curated databases. The left-hand box plot represents the distribution of matrix distances between PWMs generated using this same method. The right-hand box plot (‘different’) represents matrix distances between PWMs generated using 2 different methods: phosphosite-based and peptide-screening–based [35]. Only 13 kinases characterised in [35] have sufficient known substrates for PWM construction (therefore, n = 13). As expected, PWMs generated using different approaches are more different on average than those generated using the same method. However, in most cases, the inter-PWM distances are comparable to those found for the ‘same’ category. PWM, position weight matrix. (TIF) [file pbio.3000341.s004.tif]

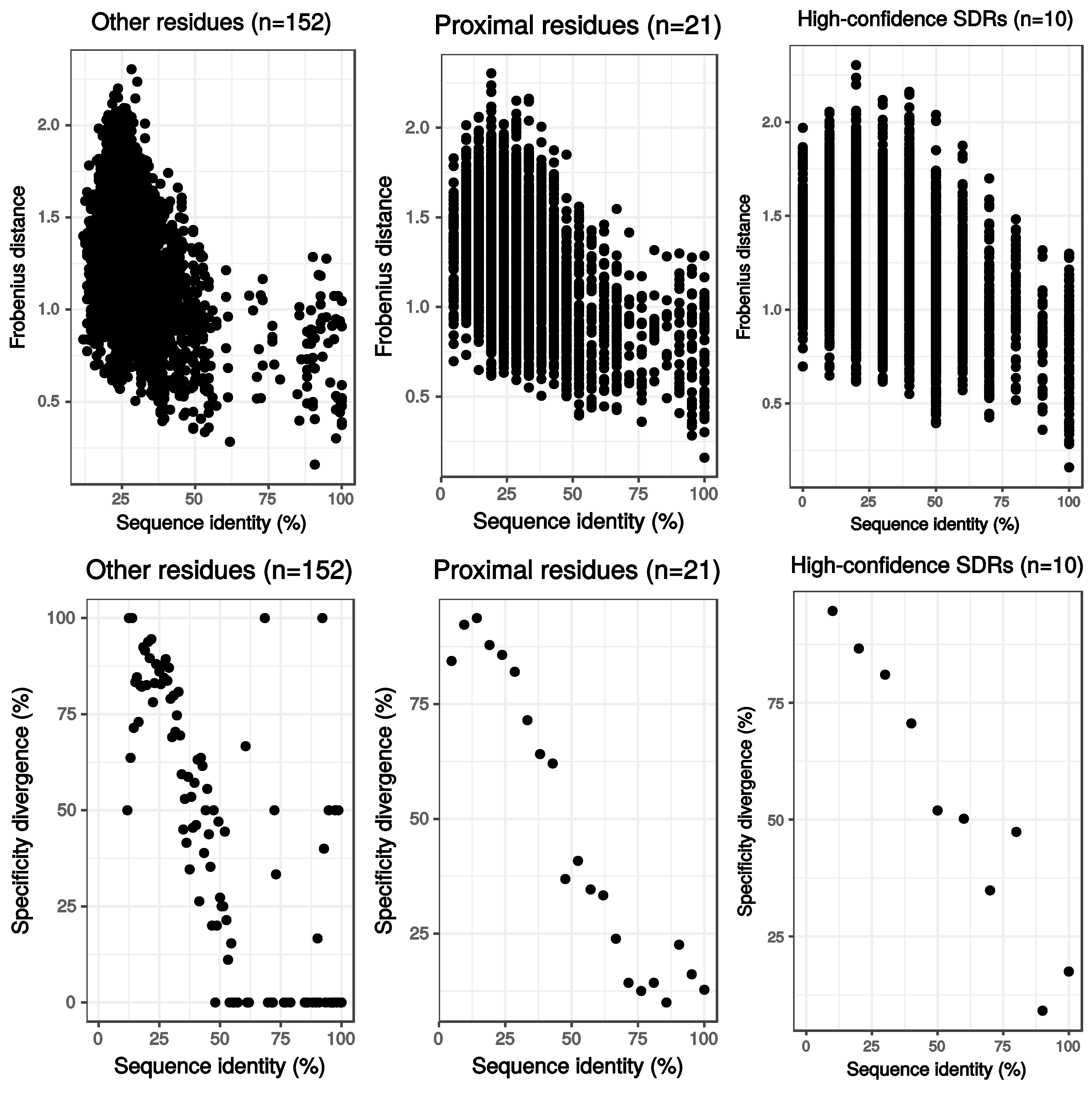

Supplement: S5 Fig — (Top) Plot between the kinase sequence identity (x-axis) and Frobenius distance (y-axis) for all possible kinase-kinase pairs among the 101 S/T kinases for which specificity models have been constructed. This has been plotted for residues of the ‘other’ category (left), ‘proximal’ category (centre), and 10 high-confidence SDRs (right) identified in [17]. (Bottom) For the same residue descriptions, plot of kinase sequence identity (x-axis) against the specificity divergence (y-axis). ‘Specificity divergence’ represents the proportion of Frobenius distances above the 1.06 threshold used to separate ‘specificity-conserved’ kinase–kinase pairs from ‘specificity-diverged’ kinase–kinase pairs (see Materials and methods). SDR, specificity determining residue; S/T, serine/threonine. (TIF) [file pbio.3000341.s005.tif]

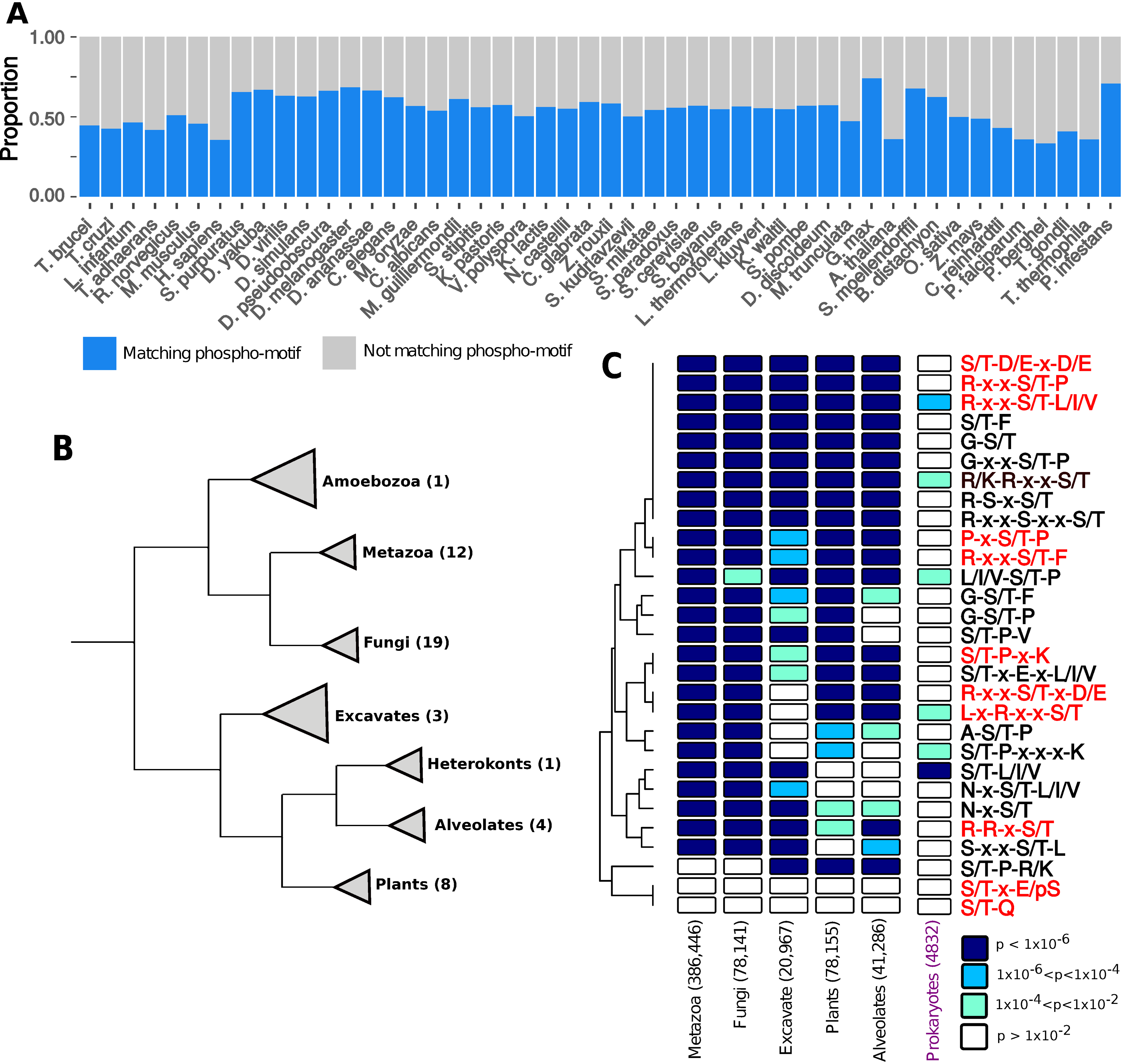

Supplement: S6 Fig — (A) Proportion of phosphorylation sites in each species that match a phosphorylation motif (see Materials and methods). (B) A simplified version of the eukaryotic tree of life presented in the [85] study. The numbers in brackets correspond to the number of different species represented by phosphorylation data in this study. (C) Calculation of binomial p-values (as in Fig 4) for each motif in each major clade (metazoa, fungi, plants, etc.) after phosphorylation sites within a clade were pooled across species. The figure legend (bottom right) is the same as in Fig 4. (TIF) [file pbio.3000341.s006.tif]

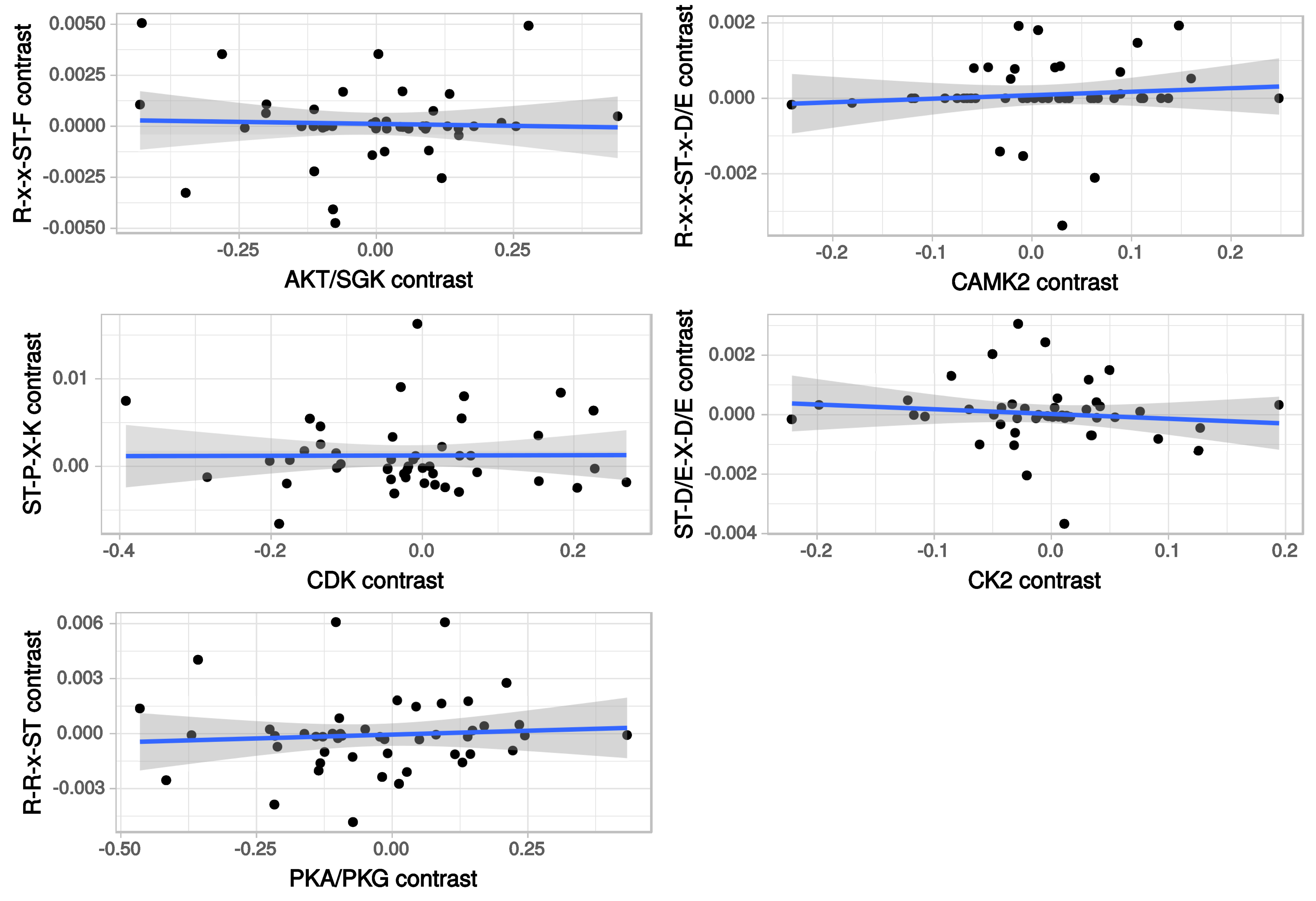

Supplement: S7 Fig — PICs between 5 different kinase clades (AKT/SGK, CAMK2, CDK, CK2, PKA/PKG) and their corresponding substrate motifs (R-x-x-S/T-F, R-x-x-S/T-x-D/E, S/T-P-x-K, S/T-D/E-x-D/E, and R-R-x-S/T, respectively). This approach accounts for the phylogenetic nonindependence between data points when comparing 2 continuous variables [50]. CAMK2, Calcium/calmodulin-dependent protein kinase type 2; CDK, Cyclin-Dependent Kinase; CK2, Casein Kinase 2; PIC, phylogenetic independence contrast; PKA, Protein Kinase A; PKG, Protein Kinase G; SGK, Serum and Glucocorticoid-regulated Kinase. (TIF) [file pbio.3000341.s007.tif]

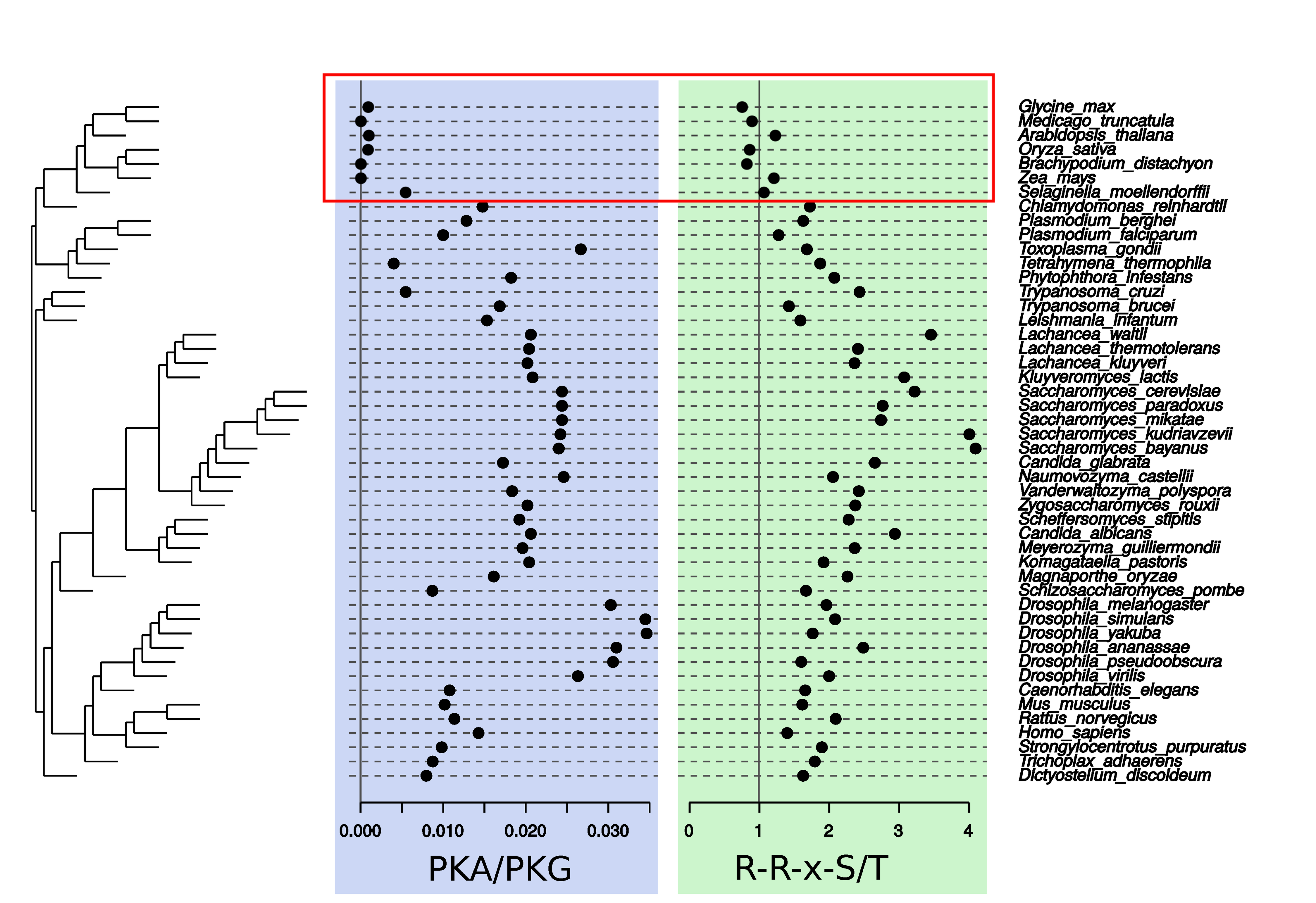

Supplement: S8 Fig — Relative kinase frequencies across the 48 species were calculated for the PKA/PKG family, and motif enrichments were calculated for their cognate substrate motif (R-R-x-S/T). The red box highlights species where the absence of PKA and PKG kinases in the proteome corresponds to a lack of R-R-x-S/T motif enrichment. PKA, Protein Kinase A; PKG, Protein Kinase G. (TIF) [file pbio.3000341.s008.tif]

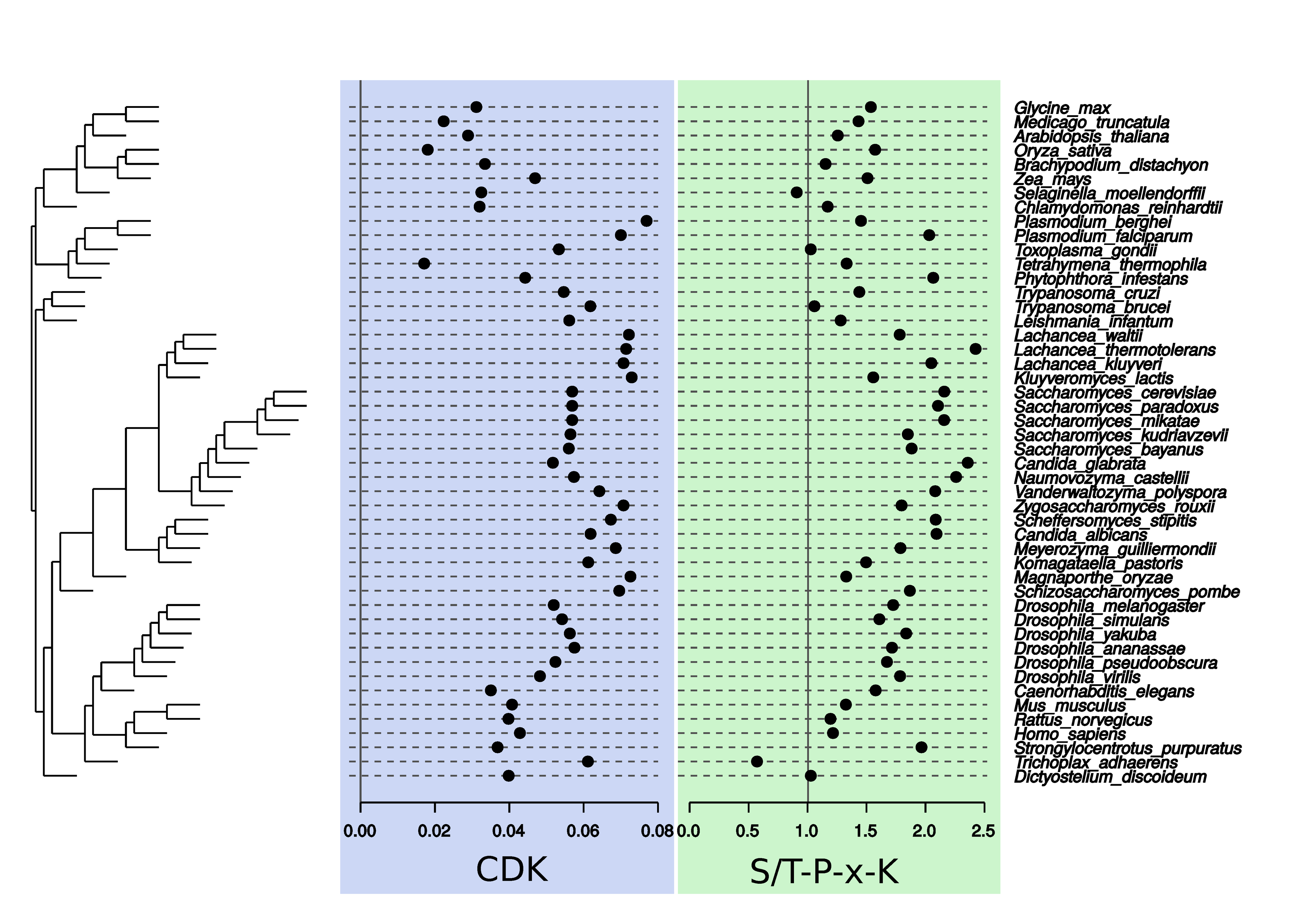

Supplement: S9 Fig — Relative kinase frequencies across the 48 species were calculated for the CDK family, and motif enrichments were calculated for its cognate substrate motif (S/T-P-x-K). CDK, Cyclin-Dependent Kinase. (TIF) [file pbio.3000341.s009.tif]
